# Integrative Multi-Omics Analysis of Multiple Sclerosis Reveals Cell-Type-Specific Regulatory Landscapes and Discordant Methylation–Expression Coupling

**DOI:** 10.3390/ijms27167275

**Published:** 2026-08-14

**Authors:** Alper Bülbül, Özdeyiş Hülya Yılmaz-İşgördü, Meziyet Dilara Reda, Eda Tahir Turanlı

**Affiliations:** 1 Department of Biostatistics and Bioinformatics, Graduate School of Health Sciences, Acibadem Mehmet Ali Aydinlar University, 34638 Istanbul, Turkey; abdullah.bulbul@live.acibadem.edu.tr; 2 Department of Molecular Biology and Genetics, Faculty of Engineering and Natural Sciences, Acibadem Mehmet Ali Aydinlar University, 34638 Istanbul, Turkey; ozdeyis.isgordu@acibadem.edu.tr; 3 Department of Molecular Biology and Genetics, Graduate School of Natural and Applied Science, Acibadem Mehmet Ali Aydinlar University, 34638 Istanbul, Turkey; meziyet.reda@live.acibadem.edu.tr

**Keywords:** multiple sclerosis, multi-omics integration, DNA methylation, single-cell RNA sequencing, proteomics, network biology

## Abstract

Multiple sclerosis (MS) is an immune-mediated central nervous system disease with mapped genetic risk loci but unresolved cross-layer regulatory mechanisms. We harmonised 30 primary public MS datasets spanning bulk transcriptomics (552 samples, 367 MS/185 healthy control [HC]), DNA methylation arrays (475 samples, 244 MS/231 HC) with sorted-cell whole-genome bisulfite sequencing (WGBS) assessment, single-cell RNA sequencing (RNA-seq; 517,533 cells from 81 donors), and cerebrospinal fluid (CSF) and brain white-matter proteomes, and UK Biobank Pharma Proteomics Project (UK Biobank-PPP) plasma proteomic statistics (407 MS/39,979 HC) for external validation. We prioritised MS-associated genes showing inverse-concordant, cohort-level methylation–expression associations and orthogonal support across layers. After harmonised reprocessing and per-stratum differential testing, a bulk-RNA × methylation inverse-concordance filter followed by an independent proteomic and/or donor-level single-cell anchor identified two Tier-1 candidates (*IKZF1*’s anchor is a candidate-panel-adjusted donor-level pseudobulk association, not transcriptome-wide significance): *ITGB2* and *IKZF1*. *ITGB2* showed the strongest cross-layer support: RNA down-regulation, promoter hypermethylation and reduced plasma abundance in UK Biobank MS cases, a pattern consistent with altered leukocyte-integrin adhesion. Pathway and network analyses linked the candidates predominantly to leukocyte and lymphocyte activation and differentiation, leukocyte-integrin adhesion, and interferon and cytokine-signalling immune modules. These findings nominate MS-relevant regulatory candidates and provide a reusable multi-omics framework for hypothesis generation and validation.

## 1. Introduction

Multiple sclerosis (MS) is a complex immune-mediated demyelinating disease of the central nervous system (CNS) that affects approximately 2.8 million individuals worldwide [1] and is among the leading causes of non-traumatic neurological disability in young adults. Its clinical course is markedly heterogeneous: the transition from relapsing-remitting to progressive disease is only loosely demarcated and remains poorly resolved at the molecular level [2], and a substantial fraction of patients continue to accumulate disability independently of clinical relapses despite high-efficacy disease-modifying therapy (progression independent of relapse activity) [3]. Decades of twin and family studies established a polygenic architecture for the disease that is now best summarised by the genome-wide susceptibility map of the International Multiple Sclerosis Genetics Consortium (IMSGC), an international collaboration that has assembled and meta-analysed the large case-control genotyping cohorts underpinning the field [4]. That reference map resolved 200 autosomal susceptibility variants outside the major histocompatibility complex (MHC) and 32 independent variants within the MHC and prioritised more than 500 candidate susceptibility genes, with statistical enrichment pointing predominantly to peripheral lymphocytes, monocytes, and microglia rather than to CNS-resident neuronal populations.

Despite this genetic resolution, the prevailing analytical practice has been to interrogate a single molecular layer in isolation: bulk peripheral-blood transcriptomics in one study [5], epigenome-wide DNA-methylation profiling in another [6], and cerebrospinal fluid (CSF) proteomics in a third [7].

Three converging developments now make a fundamentally different strategy tractable. First, the public sequence and array repositories curated by the NCBI Gene Expression Omnibus (GEO) and the EMBL-EBI archives have accumulated dozens of MS transcriptomic and methylomic series amenable to uniform reprocessing, with treatment annotations (including dimethyl fumarate and ocrelizumab arms) and, in several cases, matched cell-type information. Second, two contemporary high-depth proteomic resources have become available: a large CSF proteome quantified on the Orbitrap Astral and timsTOF platforms by Bader and colleagues [8], and a region-resolved white-matter brain proteome from progressive MS lesions [9]. Third, single-cell and single-nucleus RNA sequencing atlases of MS brain [10,11], blood, and CSF [12,13,14] now permit bulk findings to be examined directly in the cell types that express the gene of interest. Together, these resources enable a coordinated, multi-layer interrogation that no single-layer study can support.

The biological rationale for prioritising inverse-concordant methylation–expression associations, defined throughout as directionally discordant cohort-level changes between methylation and expression in the same tissue stratum, warrants explicit framing. In a homeostatic regulatory state, promoter hypermethylation and transcriptional silencing are coupled, with methylation acting as a stable, heritable brake on transcription [15,16]. A coordinated change in which promoter methylation and transcript abundance move in opposite directions (hypermethylation with transcriptional down-regulation, or hypomethylation with up-regulation) marks a candidate regulatory perturbation coupling the two layers. The design fixes the direction of the coupling but not the direction of causation, which is taken up in the Discussion; what it identifies is a locus at which regulatory and output layers move apart, the kind of methylation-linked dysregulation described conceptually in other disease settings [17]. By contrast, same-direction co-movement of methylation and transcript (both up or both down) is more readily explained by cellular-composition shifts, replication-associated demethylation or shared upstream cytokine patterns. By scanning explicitly for inverse-concordant pairs across multiple matched tissues, we concentrate the candidate list on genes whose regulatory and output layers move in opposite directions in MS-relevant cell types. Immune-cell composition is a well-recognised confounder that has historically inflated bulk-expression candidate lists in this disease, and the requirement may down-weight the subset of composition-driven signal that moves methylation and transcript in the same direction; it does not, however, eliminate cell-composition confounding, because a shift in immune-cell proportions can equally generate an inverse pairing. The filter is therefore a prioritisation heuristic and not a control for composition, a limitation returned to in Section 3.

Building on these resources and this rationale, we hypothesised that disease-relevant MS genes can be prioritised more reliably by combining inverse-concordant methylation–expression associations at the bulk level with proteomic anchoring in the relevant biofluid or tissue and donor-level single-cell validation in the originating cell type, with network-level functional context used for interpretation rather than for selection. Genes whose inverse-concordant bulk signal is joined by independent proteomic and/or donor-level single-cell support are correspondingly less likely to reflect processing artefacts and more likely to be biologically interpretable; whether any of them is therapeutically actionable is a separate question this design does not address.

The present manuscript implements this strategy end-to-end. We assemble and harmonise four omic layers across 30 datasets (one further transcriptomic series contributes to the harmonised expression matrix but not to the differential analyses), scan systematically for inverse-concordant methylation–expression pairs, anchor each surviving candidate in independent proteomic and/or donor-level single-cell evidence, embed the resulting set in a STRING v12 [18] physical-interaction network with pathway-level g:Profiler enrichment [19], and cross-reference each Tier-1 candidate against the MS genome-wide association study (GWAS) reference map. The pipeline is documented in the Data Availability section and is designed to be reusable for any disease in which inverse-concordant methylation–expression associations are biologically expected.

Our contributions are threefold. (i) We deliver a curated, batch-corrected MS multi-omics resource that can be re-queried for any candidate gene. (ii) We integrate four orthogonal omic layers (bulk transcriptomics, DNA methylation, proteomics and single-cell) with protein–protein-network context to anchor each inverse-concordant candidate in independent proteomic and/or donor-level single-cell evidence. (iii) Applying a strict bulk-RNA × methylation inverse-concordant filter, we identify two Tier-1 candidates: *ITGB2* and *IKZF1*. *ITGB2* showed the strongest cross-layer support, with lower plasma abundance in UK Biobank in the same direction as its RNA down inverse-concordant call. *IKZF1* connects IMSGC-implicated peripheral-immune and B-cell-lineage biology [4] to cereblon-modulator immunomodulatory drugs that degrade IKZF1 [20,21].

## 2. Results

### 2.1. Overview of the Integrated Pipeline

**Figure 1:**
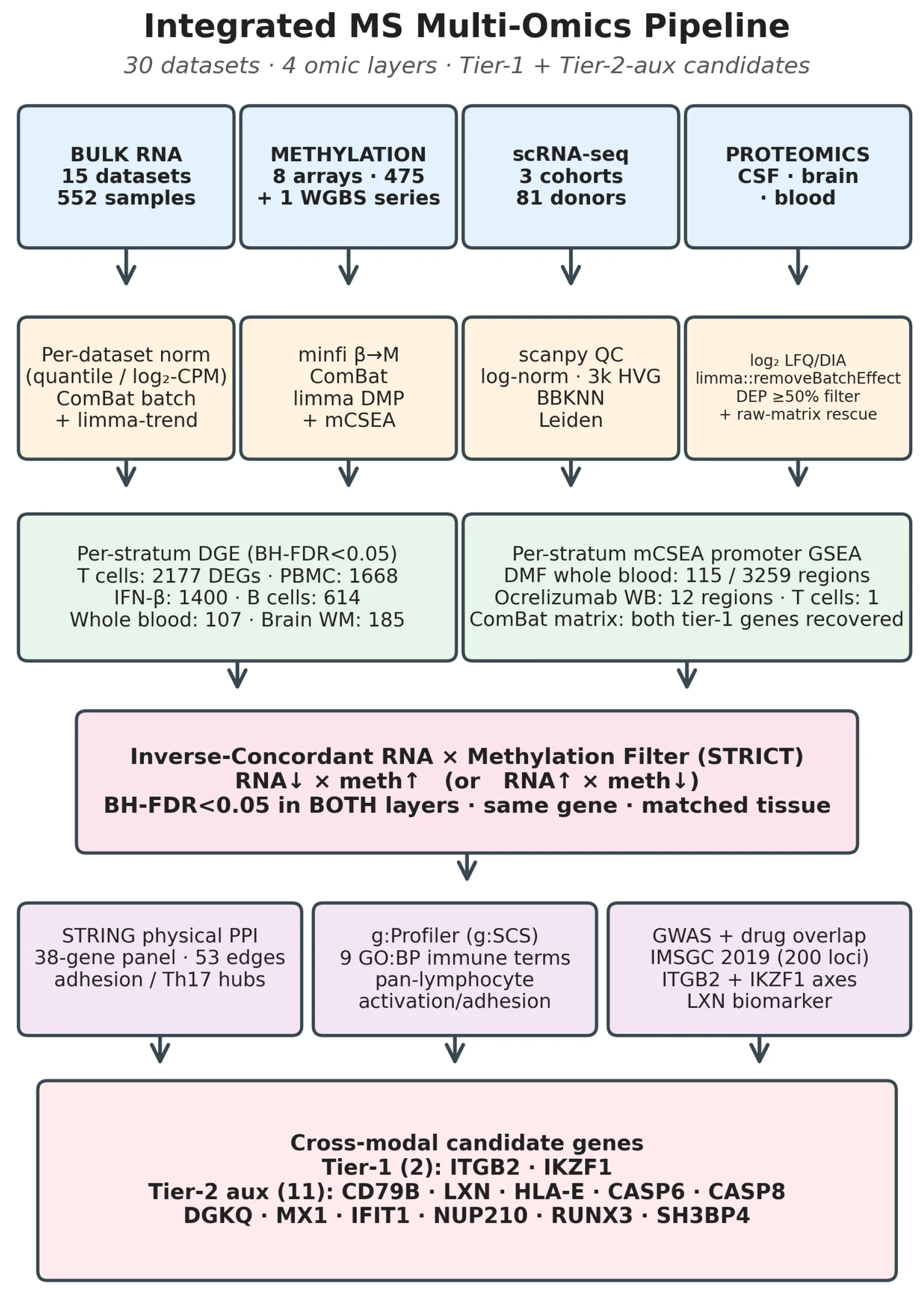
Four-track multi-omics analytical workflow. Bulk transcriptomic, bulk methylation and proteomic discovery layers (three of the four tracks) are jointly batch-corrected by dataset (GSE/cohort) with the multiple sclerosis (MS) vs. control covariate retained and tested with limma-based modelling. Single-cell RNA sequencing data (the remaining track) are processed independently and used as a per-tissue validation layer. The cross-omics integration step requires inverse methylation × RNA concordance plus an independent proteomic and/or donor-level single-cell anchor. The final stage performs STRING v12 [18] physical protein–protein interaction analysis and g:Profiler [19] functional enrichment, with each Tier-1 candidate additionally cross-referenced against the MS genome-wide association study (GWAS) susceptibility map.

Figure 1 summarises the multi-layer pipeline. Bulk transcriptomic and methylation tracks pass through per-platform normalisation, joint ComBat [22] batch correction (with MS vs. control retained as a model covariate) and differential testing (limma-trend [23] and limma differentially methylated position (DMP) + methylated CpG site enrichment analysis (mCSEA) [24], respectively). The proteomic track is harmonised within cohort with log_2_ label-free quantification (LFQ)/data-independent acquisition with parallel accumulation–serial fragmentation (DIA-PASEF) intensity normalisation, batch-corrected with limma::removeBatchEffect [23], and tested with the Differential Enrichment analysis of Proteomics data (DEP) package. The single-cell track is not batch-corrected at the bulk level: each cohort is processed independently and treated as a validation layer rather than a discovery layer, with cell-type-specific tie-corrected Wilcoxon tests. All four tracks converge on the cross-omics integration node, where the tier rule is applied. A gene is called inverse-concordant when its bulk-RNA and DNA-methylation effects are both significant at a Benjamini–Hochberg false discovery rate (BH-FDR) < 0.05 within the same tissue stratum and point in opposite directions; an inverse-concordant gene is promoted to Tier-1 only if it additionally carries an orthogonal anchor, defined as a significant proteomic effect in at least one of the seven proteomic compartments and/or a significant donor-level (pseudobulk) single-cell effect, so that at least three of the four layers agree, whereas inverse-concordant genes lacking such an anchor are held at Tier-2 auxiliary. Cell-level single-cell significance is inflated by the number of cells and counts as directional support only, never as an anchor. Applying this rule yields two Tier-1 candidates, anchored in plasma proteomics (*ITGB2*) and in donor-level peripheral blood mononuclear cell (PBMC) pseudobulk (*IKZF1*), respectively. The integrated candidate list then feeds STRING v12 [18] physical-interaction network and g:Profiler [19] pathway enrichment analysis.

### 2.2. Per Cell-Type/Tissue Differential Expression

**Figure 2:**
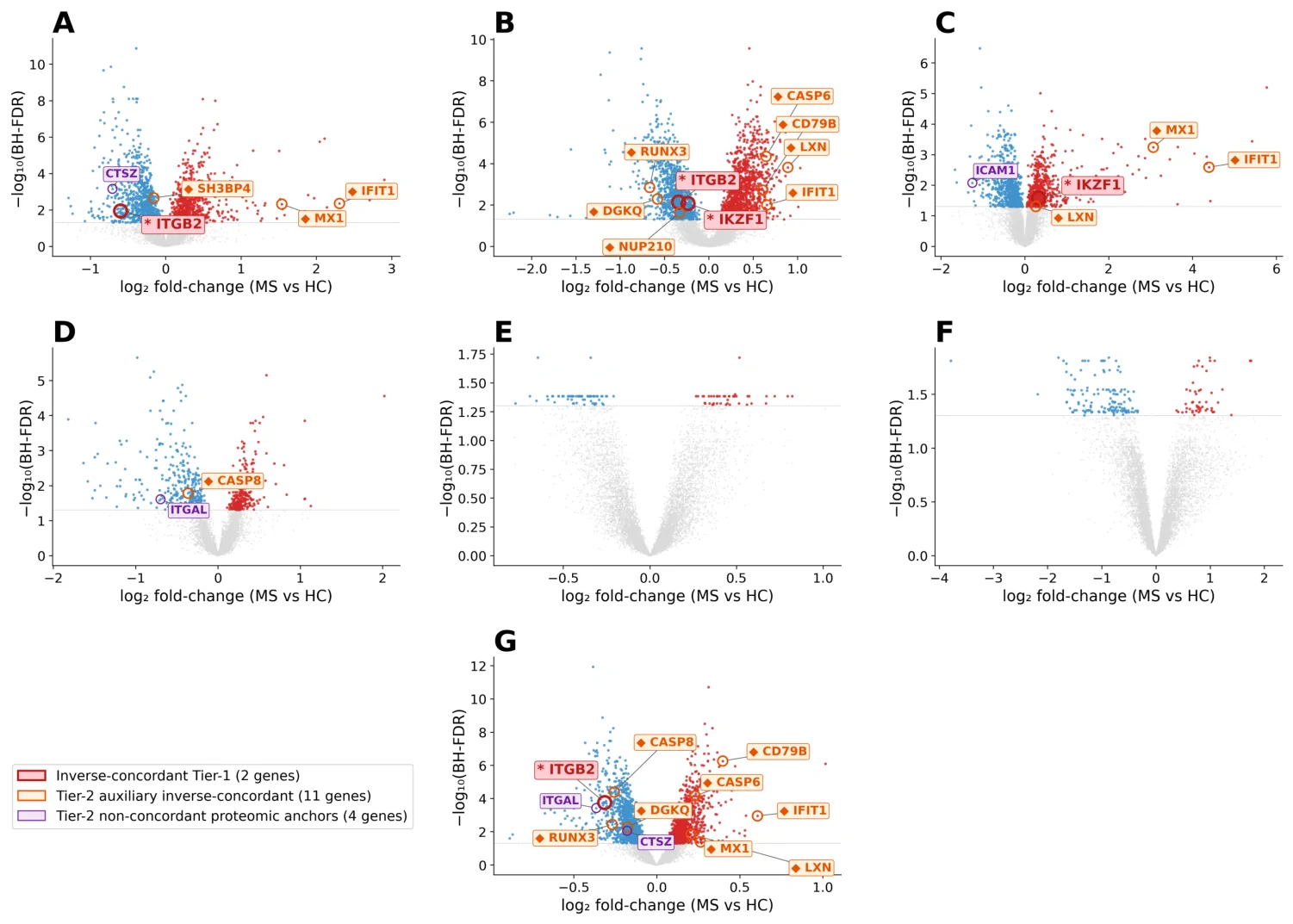
Per-stratum RNA differential expression. Seven volcano plots show limma-trend log_2_FC and −log_10_(BH-FDR): (**A**) PBMC, (**B**) T cells, (**C**) IFN-β PBMC treatment response (not MS vs. control), (**D**) B cells, (**E**) whole blood, (**F**) brain WM and (**G**) the tissue-adjusted pan-tissue case-control analysis (14 datasets, 514 samples). Each dot is one gene. Red dots are genes significantly up-regulated and blue dots genes significantly down-regulated in the contrast of that panel at BH-FDR < 0.05 (MS vs. control in all panels except (**C**), where the contrast is IFN-β vs. baseline); grey dots are genes that do not reach significance, and the dotted horizontal line marks the BH-FDR = 0.05 threshold (−log_10_(BH-FDR) = 1.3). Evidence groups are encoded consistently across figures: Tier-1 red, Tier-2 auxiliary inverse-concordant orange, and Tier-2 non-concordant proteomic anchors purple. Within the gene labels, an asterisk (*) marks an inverse-concordant Tier-1 gene and a filled rhombus (♦) a Tier-2 auxiliary inverse-concordant gene. Only BH-FDR-significant candidates are labelled.

We first inspected the per-stratum differential expression landscape (Figure 2). Per-stratum analysis revealed strong cell-type specificity in the number of differentially expressed genes (DEGs) at BH-FDR < 0.05. All contrasts are MS vs. control unless marked otherwise, and the T-cell stratum includes GSE137143, whose CD4 and CD8 fractions come from the same 26 donors and whose control arm is a single donor, so the dataset is modelled as a batch covariate there (Section 4.2). T cells gave 2177 DEGs (*STAT3* log_2_FC=−0.59 FDR = $7.0\times 10^{-7}$; *CASP6* +0.65 $FDR=4.5\times 10^{-5}$; *LXN* +0.89 FDR = $1.5\times 10^{-4}$; *ITGB2* −0.34 FDR = $7.3\times 10^{-3}$; *ITGAL* is trend-level here, −0.30 FDR = 0.058), and PBMC 1668 DEGs led by the canonical type-I interferon response (*MOSPD3* −0.39 FDR = $1.4\times 10^{-11}$; *RPAP2* +0.42 FDR = $2.3\times 10^{-6}$; *IFI44L* +2.90 FDR = $2.2\times 10^{-4}$; *CTSZ* −0.71 FDR = $7.0\times 10^{-4}$). The interferon-β (IFN-β)-treated PBMC treatment-response contrast (IFN-β vs. baseline, not MS-vs-control) yielded 1400 DEGs (*IFI44L* +5.77 FDR = $6\times 10^{-6}$; *MX1* +3.06 FDR = $5.8\times 10^{-4}$; *IFIT1* +4.39 FDR = $2.6\times 10^{-3}$). B cells gave 614 DEGs and whole blood 107, where Tier-1 *ITGB2* (−0.25, FDR = 0.055), *STAT3* (−0.32, FDR = 0.055) and *HLA-E* (−0.31, FDR = 0.051) were trend-level and are not counted among the 107. Brain white matter (WM) gave 185 DEGs, where *CHL1* (+0.81, FDR = 0.14) and *THRB* (+0.92, FDR = 0.14) were trend-level only, neither counted among the 185 nor passing the strict inverse-concordant filter. The brain stratum signal is constrained by sample size (*n* = 18 MS/12 healthy control [HC]) but the direction of effect is consistent with the Absinta et al. [10] chronically active lesion phenotype. A combined pan-tissue analysis pooling all five case-control strata (quantile-normalised to a common scale, ComBat-corrected by dataset, tissue-adjusted limma ~ tissue + condition; 14 datasets, 514 samples) is shown in Figure 2G. *ITGB2* retains its inverse-concordant direction and significance once all strata are pooled and tissue is adjusted for (log_2_FC = −0.314, FDR = $1.8\times 10^{-4}$), whereas the Tier-2 auxiliary gene *HLA-E* does not (log_2_FC = +0.045, FDR = 0.61); its inverse-concordant pairing is carried by the whole-blood stratum. *IKZF1* does not reach significance in the pooled analysis (FDR = 0.67); its inverse-concordant evidence derives from the T-cell stratum, and both of its cohort pairings are accordingly T-cell pairings.

### 2.3. Methylation Regulatory Landscape

**Figure 3:**
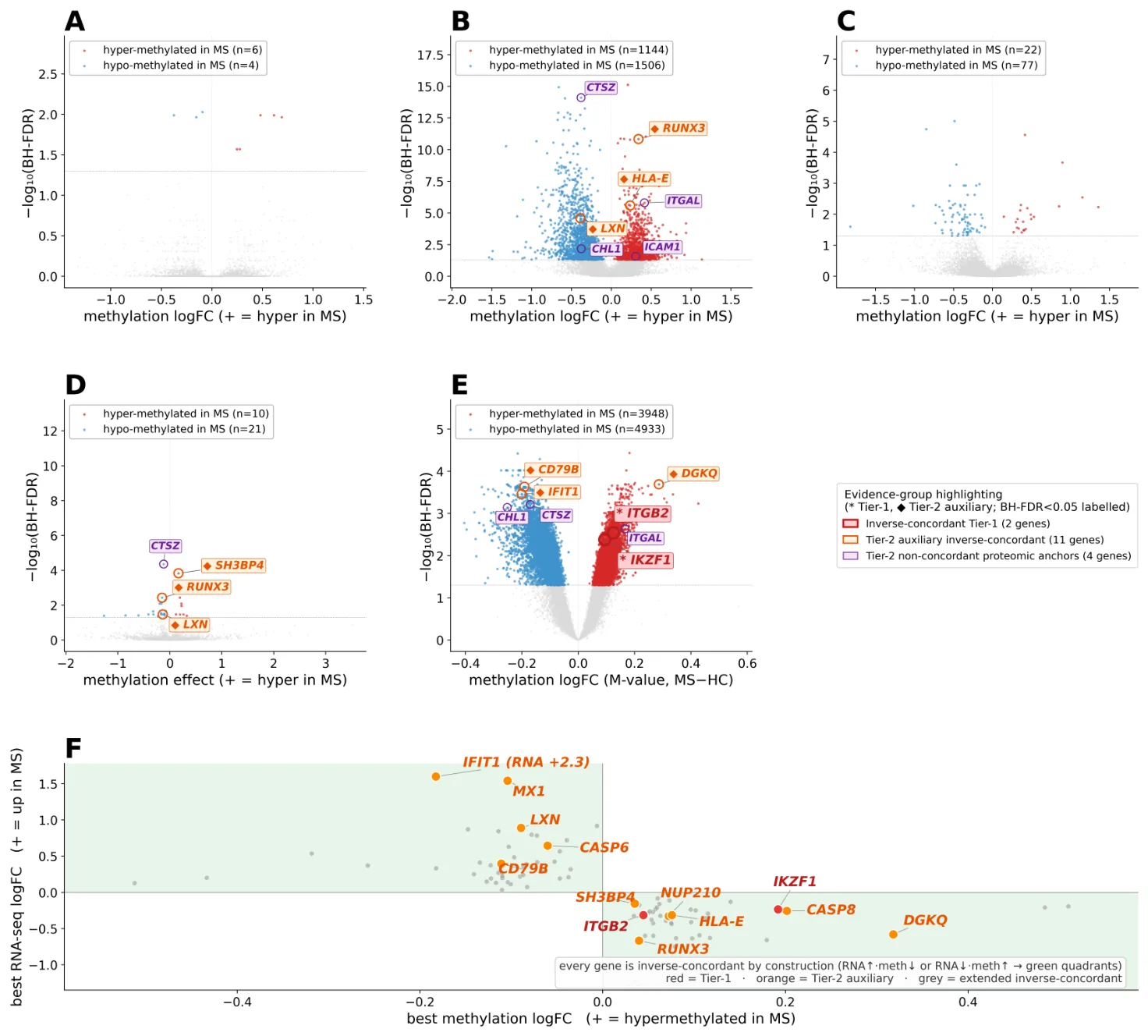
Gene-level methylation validation of MS candidate genes. (**A**–**D**) Differential-methylation volcano plots for (**A**) T-cell baseline, (**B**) DMF-treated whole blood, (**C**) ocrelizumab-treated whole blood and (**D**) brain white matter. Positive logFC denotes hypermethylation in MS. (**E**) Combined analysis of eight array datasets after preprocessing and ComBat correction. (**F**) The inverse-concordant discovery pool, in which RNA and methylation effects are significant and oppositely directed. For readability the RNA axis of this panel is capped at +1.6; the two interferon-driven genes that exceed it, *MX1* and *IFIT1*, are drawn at the cap and their true RNA log_2_FC is given in parentheses beside the gene label. Evidence-group colours are used consistently across the panels: Tier-1 genes are red and Tier-2 auxiliary inverse-concordant genes orange, and in Panel (**F**) the remaining genes of the discovery pool are grey. Tier-2 non-concordant proteomic anchors are shown in purple in Panels (**A**–**E**).

Figure 3 displays the per-stratum methylation analysis as uniform gene-level differential testing (limma-DMP) on a common methylation logFC axis; throughout, log_2_FC denotes an RNA or protein log_2_ fold change, whereas the unsubscripted logFC denotes the limma methylation contrast on M-values. The dimethyl fumarate (DMF)-treated whole-blood stratum yielded the largest number of differentially methylated genes (2650 of 16,667 at BH-FDR < 0.05), and promoter-level mCSEA [24] enrichment on the same matrix agrees (115 of 3259 promoters); this is a compartment-level check, not independent replication. The top DMF methylation anchors *THRB* (gene-level logFC =−0.59, BH-FDR = $4.7\times 10^{-7}$; mCSEA normalised enrichment score [NES] = −2.41), *CTSZ* (logFC = −0.38, FDR = $7.9\times 10^{-15}$) and *CHL1* (logFC = −0.38, FDR = $6.7\times 10^{-3}$) are all hypomethylated in MS, and none passes the joint RNA × methylation inverse-concordant filter. *CTSZ* and *CHL1* are carried forward as Tier-2 non-concordant proteomic anchors. *THRB* is not assigned to that group, because it was not quantified in any of the seven proteomic compartments and therefore cannot provide a proteomic anchor; it is reported here as a methylation-stratum finding and is excluded from the tiered panel. The ocrelizumab whole-blood stratum is more conservative (99 gene-level DMPs; 12 mCSEA promoters); the smaller count is not by itself evidence of a milder epigenetic effect, since stratum size, platform and statistical power all differ, and the treatment-free T-cell baseline stratum (*n*_MS_ = 18, *n*_HC_ = 35) is the sparsest (10 gene-level DMPs; 1 mCSEA promoter). Inverse-concordance aggregation across the whole-blood meta-cohort produced the inverse-concordant Tier-2 auxiliary genes *LXN* (best RNA FDR = $2.0\times 10^{-3}$, best meth FDR = $1.3\times 10^{-12}$), *RUNX3* (transcript-down/increased gene-level methylation) and *CASP6* (transcript-up/decreased gene-level methylation); the PBMC and pan-tissue scans further produced the Tier-2 auxiliary gene *SH3BP4* (transcript-down/increased gene-level methylation) and inverse-concordant Tier-1 *ITGB2* (transcript-down/promoter-hypermethylated, 8 cohort pairings); the Tier-2 auxiliary gene *HLA-E* showed a significant methylation arm here (increased gene-level methylation, 2 cohort pairings, meth BH-FDR = $8.1\times 10^{-8}$) with a matching bulk-RNA down-call; it is nevertheless not Tier-1, because it carries no qualifying proteomic and/or donor-level single-cell anchor (Section 2.1). *THRB*, *CTSZ* and *CHL1*, although showing strong methylation signal in the DMF stratum, do not pass the strict joint-significance inverse-concordant filter (the RNA layer does not reach FDR < 0.05 in the matched stratum). *LXN* methylation is detected in both DMF and ocrelizumab cohorts; given the size of these strata (*n* = 12–24 per arm), this is preliminary evidence that the methylation signal is preserved across treatments rather than evidence of treatment independence. Reprocessing all eight array methylation series uniformly from raw Illumina intensity data (IDAT) files (minfi preprocessIllumina, six series) or deposited signal/beta matrices (two series), merging on 270,123 common Illumina Infinium HumanMethylation450 (450K) probes and ComBat batch-correcting (475 samples, 244 MS/231 HC; batch removal confirmed by principal component analysis (PCA)), reproduced in the jointly processed matrix both inverse-concordant Tier-1 candidates, three Tier-2 auxiliary inverse-concordant genes and the Tier-2 auxiliary gene *HLA-E* at BH-FDR < 0.05 (*HLA-E* logFC = +0.10, FDR = $6.2\times 10^{-3}$; *ITGB2* +0.13, $2.9\times 10^{-3}$; *LXN* −0.18, $4.1\times 10^{-4}$; *CD79B* −0.19, $2.3\times 10^{-4}$; *IKZF1* +0.09, $4.2\times 10^{-3}$; *SH3BP4* +0.12, $1.7\times 10^{-3}$; Figure 3), confirming the methylation arm on a uniformly normalised, batch-corrected matrix.

Cell-type-resolved and orthogonal-platform methylation assessment. To test whether the array-level Tier-1 methylation signals hold at sorted-cell-type resolution and on an orthogonal sequencing platform, we examined sorted-immune whole-genome bisulfite sequencing (GSE173787; CD19 B cells, CD4, CD8 and CD14 monocytes; 72 MS/57 HC donor × cell-type profiles across the four sorted populations). At sorted-cell-type resolution, these cohorts are markedly underpowered (*n* ≈ 10–26 per arm per cell type); after per-cell-type BH-FDR correction, neither inverse-concordant Tier-1 promoter reached significance in any individual sorted cell type; among the Tier-2 auxiliary genes, *CD79B* showed a directionally consistent nominal promoter hypomethylation in B cells (Δβ = −0.014, *p* = 0.025), in keeping with its inverse-concordant call and B-lineage identity, and the strongest B-cell candidate signals were the auxiliary genes *RUNX3*, *CTSZ* and *ICAM1* (all BH-FDR < 0.05, hypomethylated in MS); *ICAM1* was the only candidate also reaching BH-FDR < 0.05 outside B cells (CD4 T cells, BH-FDR = 0.046). These orthogonal cohorts therefore complement rather than replace the array-based discovery: they corroborate the B-lineage methylation context of *CD79B* while flagging that single-cell-type and whole-blood promoter resolution is currently power-limited for the Tier-1 genes *ITGB2* and *IKZF1*.

### 2.4. Inverse-Concordant Discovery

Genes passing the inverse-concordant filter (FDR < 0.05 in both bulk RNA and methylation, with opposite effects) form a 94-gene discovery pool. The named subset comprises two Tier-1 genes (*ITGB2*, *IKZF1*) and eleven Tier-2 auxiliary inverse-concordant genes (*CD79B*, *LXN*, *HLA-E*, *SH3BP4*, *CASP6*, *CASP8*, *DGKQ*, *MX1*, *IFIT1*, *NUP210*, *RUNX3*). *LXN* has the strongest RNA–methylation signal but only nominal proteomic and direction-discordant single-cell support; it is therefore assigned to Tier-2. These 13 genes, together with four Tier-2 non-concordant proteomic anchors (*CTSZ*, *CHL1*, *ICAM1*, *ITGAL*), make up the 17 tiered genes carried through the remainder of the analysis. The strict enrichment panel and the cross-modal display therefore contain the same 17 tiered genes, and these 17 candidates are embedded with 21 connected canonical MS markers in a 38-gene network; the two displays are presented with their respective results in Section 2.6 and Section 2.7.

Because the gene-level methylation statistic aggregates promoter and gene-body probes, and gene-body methylation often correlates positively with transcription, each of the 94 genes was classified by the compartment supporting its methylation call (Supplementary Table S2). Only eight genes (9%) are anchored by mCSEA promoter-region enrichment, while the remaining 86 (91%) rest on the promoter-plus-gene-body composite alone. Restricting the filter to mCSEA-significant promoter regions exclusively therefore retains 8 of the 94 genes: both Tier-1 candidates, *ITGB2* (promoter q=0.016, significant in PBMC, T cells, whole blood and the pan-tissue meta-summary) and *IKZF1* (q=0.030; T cells); two of the eleven Tier-2 auxiliary genes (*LXN*, q=0.0024, the strongest promoter signal in the pool, in T cells, pan-tissue and the IFN-β PBMC contrast; and *CASP8*, q=0.019; pan-tissue), and four genes from the extended pool (*LIME1*, *KCNB1*, *PRDM1*, *ZAP70*). The Tier-2 auxiliary candidate *CD79B* fails this stricter definition for a technical rather than a biological reason, and for this gene probe-level and region-level promoter evidence must be kept apart. At probe level, the promoter evidence exists: *CD79B* carries three promoter-annotated probes on the 450K/EPIC (Illumina Infinium MethylationEPIC) intersection, all hypomethylated in MS in the same direction, and these promoter probes are what drives the gene-level aggregate underlying its inverse-concordant call (T cells and the pan-tissue meta-summary). At region level, no test exists at all: three probes fall below the minimum region size required by mCSEA, so no promoter-region enrichment statistic could be constructed for the gene. *CD79B* is therefore absent from the promoter-anchored set because its promoter compartment could not be assessed, not because it was assessed and rejected, and its promoter support should be read as probe-level support that was never subjected to region-level testing. *SH3BP4*, by contrast, was testable at the promoter (11 probes) but did not reach significance there (q=0.18) and its signal is gene-body-weighted in the dimethyl-fumarate stratum; this compartment ambiguity is one of the reasons it is placed at Tier-2 (Section 3). The remaining six Tier-2 auxiliary genes (*CASP6*, *DGKQ*, *MX1*, *IFIT1*, *NUP210*, *RUNX3*) do not survive the promoter-only definition either. The discovery pool is therefore best regarded as a hypothesis-generating set in which promoter attribution is established for a minority of genes. Four of the eight promoter-anchored genes are tiered candidates, namely both inverse-concordant Tier-1 genes (*ITGB2*, *IKZF1*) and two Tier-2 auxiliary genes (*LXN*, *CASP8*); the remaining four (*LIME1*, *KCNB1*, *PRDM1*, *ZAP70*) come from the extended pool. These four tiered genes are the candidates for which the strict promoter-silencing rationale underlying the inverse-concordant prioritisation is directly supported, and no further subset of them is privileged here.

### 2.5. Proteomic Validation

**Figure 4:**
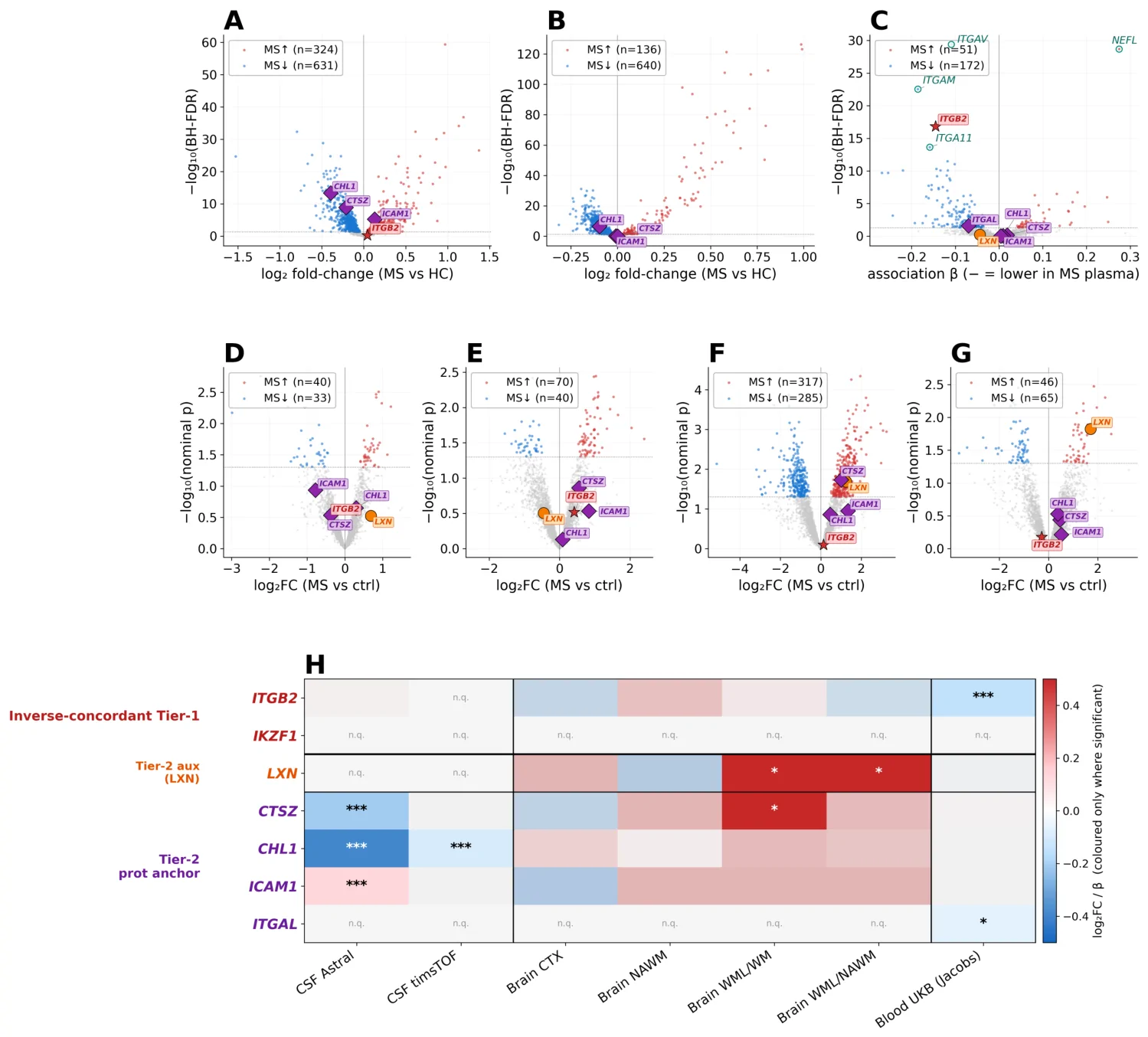
Proteomic validation across CSF, brain, and blood. (**A**,**B**) Volcano plots of re-analysed Bader & Mann CSF DIA-MS data from Astral and timsTOF platforms. (**C**) UK Biobank-PPP Olink plasma associations (407 prevalent MS vs. 39,979 controls), highlighting ITGB2, leukocyte-integrin markers, and NEFL. (**D**–**G**) Region-resolved Wang & Julien brain proteome comparisons across cortex, NAWM, WML vs. control WM, and WML vs. MS-NAWM. In Panels (**A**–**G**), small red and blue dots are proteins significantly higher and lower in MS at the significance threshold of that panel, small grey dots are proteins that do not reach it, and the dotted horizontal line marks the threshold. Candidate proteins are drawn as enlarged markers coloured by evidence group: the inverse-concordant Tier-1 genes *ITGB2* as a red star, the Tier-2 auxiliary inverse-concordant gene *LXN* as an orange circle, and the four Tier-2 non-concordant proteomic anchors (*CTSZ*, *CHL1*, *ICAM1*, *ITGAL*) as purple diamonds. The green open circles in Panel (**C**) mark the remaining top-ranked plasma proteins of that cohort (ITGAV, ITGAM, ITGA11 and NEFL). (**H**) Directional-consistency heatmap summarising significant proteomic effects across CSF, brain, and blood compartments. Colours indicate log_2_FC or plasma association β; faded cells indicate non-significant measured effects, and n.q. indicates not quantified or below the cohort’s completeness threshold. Asterisks grade the significance of the effect in that compartment (* *p* < 0.05; *** *p* < 0.001) and are computed on the BH-FDR for the two CSF columns and the blood column, and on the nominal *p*-value for the four brain columns, in which no protein survives BH correction. In all volcano panels (**A**–**G**), the legend *n* is the number of proteins significantly higher (MS↑) or lower (MS↓) in MS at the panel’s significance threshold, not the sample size.

Figure 4 places the candidate set onto the available proteomic compartments, each re-analysed from primary or summary-statistic data. Two CSF datasets come from the Bader & Mann 2026 cohort on two instruments: (A) the Astral data-independent acquisition mass spectrometry (DIA-MS) platform (978 MS/306 HC; 955 of 2046 quantified genes significant at BH-FDR < 0.05) and (B) the larger timsTOF DIA CSF sample set from the same study (1536 MS/2363 HC; 776 of 1069 quantified genes significant at BH-FDR < 0.05); these are two differently-sized CSF sample sets from one study profiled on two instruments. Among the two inverse-concordant Tier-1 candidates, ITGB2 is the only one quantified in CSF, and it shows no significant MS vs. control difference there (Astral log_2_FC = +0.046, FDR = 0.55). On the Astral instrument, where it is quantified, ITGB2 is the least completely measured of the candidates, detected in 71.6% of MS but only 63.1% of control samples. On the timsTOF instrument, ITGB2 falls far below the completeness threshold, quantified in only 40 MS and 32 control samples (1.8%) and therefore excluded from the tested set, where its point estimate is a non-significant log_2_FC = −0.028. Neither CSF instrument is therefore informative for it, and its proteomic anchoring rests on plasma (Figure 4C). IKZF1 is not quantified in any of the seven proteomic compartments (neither CSF instrument, none of the four brain-region contrasts and none of the 2911 UK Biobank-PPP plasma assays), and is absent even from the pre-filter intensity matrices, so its absence reflects non-detection rather than the completeness threshold. Its Tier-1 status therefore rests entirely on the donor-level single-cell anchor, and its row is accordingly marked n.q. across all compartments in Figure 4. The CSF signals come from three of the four Tier-2 non-concordant proteomic anchors, CTSZ (Astral FDR = $1.3\times 10^{-9})$, CHL1 (Astral FDR = $4.1\times 10^{-14}$; timsTOF FDR = $3.6\times 10^{-7}$) and ICAM1 (Astral FDR = $5.6\times 10^{-6}$), all three of which are quantified in every sample and are therefore unaffected by the missing-value treatment. (C) The blood compartment is the Jacobs et al. 2024 UK Biobank Pharma Proteomics Project (UK Biobank-PPP) Olink plasma study [25] (published per-protein case-control association statistics; 407 MS/39,979 HC, 2911 proteins; 223 at FDR < 0.05). ITGB2 (inverse-concordant Tier-1) is the fourth-most-significant protein in the entire UK Biobank MS plasma proteome (β = −0.145, FDR = $1.5\times 10^{-17}$, lower in MS plasma). Two of the four top-ranked proteins (ITGAV, NEFL, ITGAM, ITGB2) are subunits of the leukocyte β2-integrin axis that ITGB2 anchors, namely ITGAM and ITGB2 itself; the other two are not, ITGAV being an αV subunit that does not pair with ITGB2 and NEFL a marker of neuroaxonal damage rather than of adhesion and an established MS biofluid biomarker [26]. The fourth Tier-2 non-concordant proteomic anchor, ITGAL, is also significant (β = −0.070, FDR = $2.5\times 10^{-2}$). This is independent 40,000-participant confirmation of the ITGB2 signal, MS-down in plasma consistent with the RNA down arm of the inverse-concordant call. The brain proteomic dataset was the Wang & Julien 2026 [9] region-resolved white-matter dataset (*n* = 8 per group except white-matter lesions, *n* = 5): four region comparisons are shown ((D) cortex; (E) normal-appearing white matter (NAWM); (F) white-matter lesion vs. control WM; (G) lesion vs. MS-NAWM). At *n* = 5–8 per group, no protein survives BH-FDR < 0.05 in any brain region (73–602 proteins per region reach nominal p<0.05); of the candidates, only LXN reaches nominal significance, up in white-matter lesions on both lesion contrasts (lesion vs. MS-NAWM log_2_FC = +1.70, *p* = 0.015; lesion vs control WM log_2_FC = +1.24, *p* = 0.021), while ITGB2 does not (cortex log_2_FC = −0.34, *p* = 0.27), but the brain parenchymal proteome is underpowered and is reported as nominal-direction context only. (H) A directional-consistency heatmap summarises the two inverse-concordant Tier-1 genes, the Tier-2 auxiliary gene *LXN* and the four Tier-2 non-concordant proteomic anchors (*CTSZ*, *CHL1*, *ICAM1* and *ITGAL*) across all seven proteomic compartments (CSF Astral, CSF timsTOF, four Wang & Julien brain regions and the UK Biobank blood cohort). *LXN* follows the evidence-group colouring used across the other figures: it is drawn in the Tier-2 auxiliary inverse-concordant orange (filled circles) and occupies its own row block in panel H, distinguishing it from the Tier-2 non-concordant proteomic anchors in purple (diamonds) and from the Tier-1 candidates in red (stars). LXN is quantified in all four brain contrasts and in UK Biobank plasma, but falls below the completeness threshold on both CSF instruments and is therefore marked n.q. on those two platforms, which reflects the completeness threshold rather than incidental non-detection. A 30-study published serum/plasma/CSF-proteomic literature meta-cohort (reported differentially abundant protein [DAP] directions only) provides additional context but is not a uniform re-analysis. Because CSF, brain and blood are different biological compartments, we do not pool effect sizes across them; cross-compartment agreement is reported as directional consistency only. These proteomic comparisons are cross-sectional and span heterogeneous platforms, cohorts and protein-completeness thresholds; the brain parenchymal proteome in particular is underpowered (*n* = 5–8 per region), so the proteomic layer is interpreted as orthogonal corroboration rather than independent discovery.

Because *ITGB2* is both incompletely and unequally detected, and because the Bader cohort records a CSF leukocyte count for every sample, we tested directly whether CSF cellularity accounts for that asymmetry. Leukocyte counts are higher in the MS arm (median 4 versus 1 cells/μL; 38.4% versus 4.9% of samples above 5 cells/μL), and the probability of detecting ITGB2 rises with that count (logistic β = +0.23 per doubling, $p=7\times 10^{-4}$, adjusted for total CSF protein). Once leukocyte count enters the model, the effect of diagnosis on detection falls from β = +0.47 (p=0.004) to β = +0.22 (*p* = 0.21), and within each leukocyte stratum, MS and control detection rates are indistinguishable (Fisher exact p≥0.11 in every stratum with at least eight samples per arm; the >10 cells/μL stratum has only five controls and is not testable). The association is leukocyte-specific: PTPRC (CD45) behaves the same way (β = +0.23, p=0.05), whereas albumin does not (β = +0.006, p=0.98). Among the samples in which ITGB2 is measured, however, its abundance does not track cellularity (Spearman ρ = +0.07, p=0.08 in MS; ρ = +0.09, p=0.25 in controls). CSF cellularity therefore governs whether this leukocyte-surface protein crosses the detection threshold, not how much of it is present once it does.

### 2.6. Single-Cell Assessment in Brain, Blood and CSF

**Figure 5:**
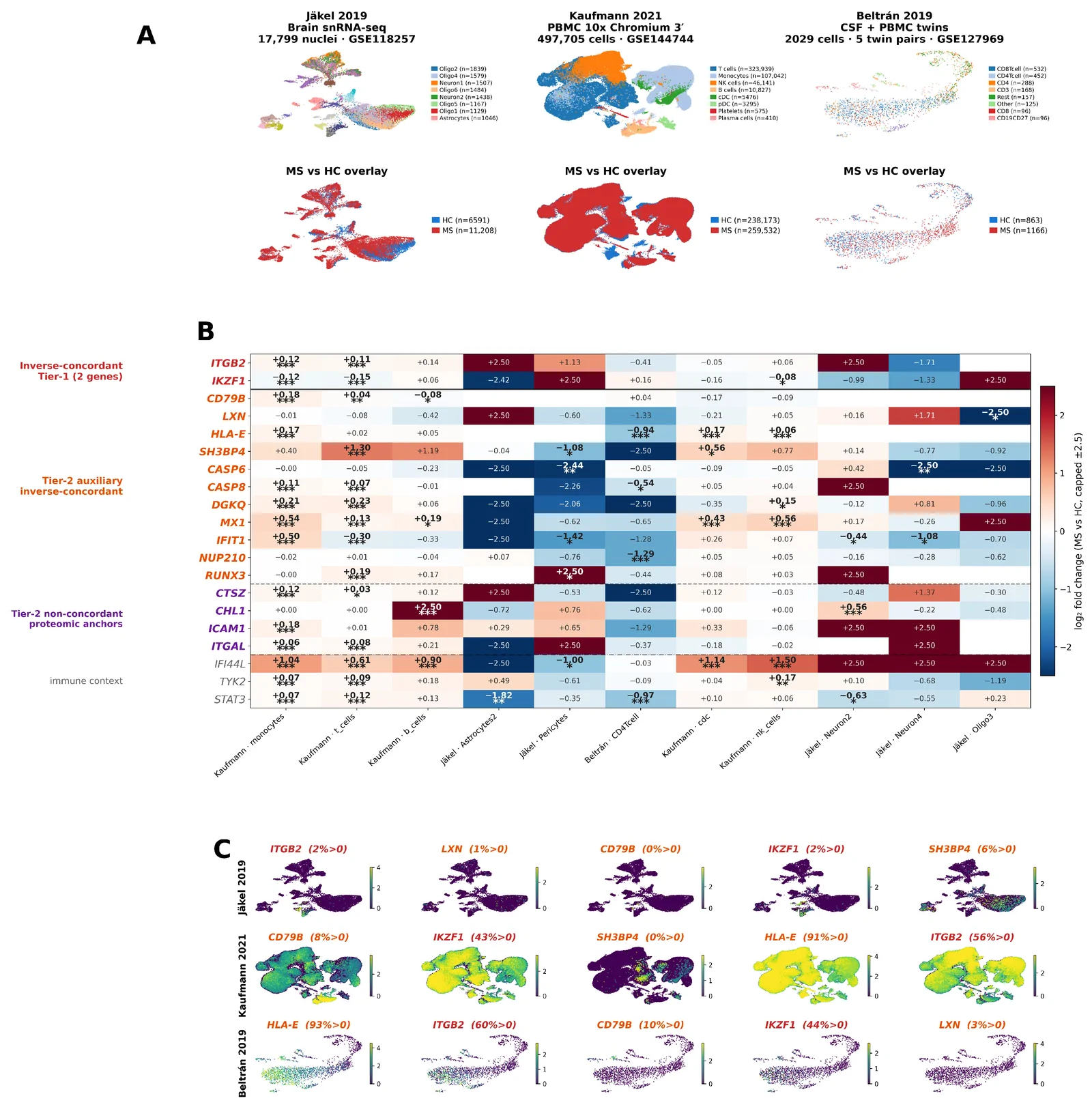
Single-cell assessment across three cohorts. (**A**) Cell-type and disease-status uniform manifold approximation and projection (UMAP) plots for Jäkel brain snRNA-seq, Kaufmann PBMC scRNA-seq and Beltrán CSF/PBMC twins. (**B**) Cell-level MS vs. control log_2_FC across testable cell types. The value printed in each cell is that log_2_FC, capped at ±2.5 as in the colour bar, and is set in bold where the effect is significant. Asterisks denote BH-FDR significance across the 783 gene × cohort × cell-type comparisons across the 25-gene testing panel (* BH-FDR < 0.05; ** BH-FDR < 0.01; *** BH-FDR < 0.001), and white cells are non-testable. Row labels use the common evidence colours: Tier-1 red, Tier-2 auxiliary inverse-concordant orange, Tier-2 non-concordant proteomic anchors purple and contextual immune genes grey. The Tier-1 genes, *CD79B*, *SH3BP4* and *HLA-E*, have direction-consistent support in at least one stratum; *LXN* is direction-discordant in Jäkel brain. These cell-level results provide directional evidence; tier assignment uses donor-level pseudobulk. (**C**) Expression overlays for selected quantified candidates; percentages indicate non-zero expression.

Three independent cohorts provided cell-type-resolved assessment: Jäkel brain single-nucleus RNA sequencing (snRNA-seq) [11], Kaufmann PBMC single-cell RNA sequencing (scRNA-seq)/cellular indexing of transcriptomes and epitopes by sequencing (CITE-seq) [13] and Beltrán discordant-twin CSF/PBMC [14] (Figure 5). Testing comprised 783 gene × cohort × cell-type comparisons across the 25-gene testing panel (*CASP6*, *CASP8*, *CD79B*, *CHL1*, *CTSZ*, *DGKQ*, *FOXP3*, *HLA-E*, *ICAM1*, *IFI44L*, *IFIT1*, *IKZF1*, *ITGAL*, *ITGB2*, *LXN*, *LYN*, *MOSPD3*, *MX1*, *NUP210*, *RPAP2*, *RUNX3*, *SH3BP4*, *STAT3*, *THRB* and *TYK2*), of which the 17 displayed candidates are the visualised subset; 114 of those comparisons were significant after BH correction. Direction-consistent effects included *ITGB2* down-regulation in Beltrán CSF/blood (log_2_FC = −2.56, FDR = $8.2\times 10^{-5}$), *IKZF1* down-regulation in Kaufmann T cells and Jäkel microglia/macrophages, *SH3BP4* down-regulation in Jäkel neuronal/vascular populations, and modest *CD79B* up-regulation in Kaufmann monocytes and T cells. Because cell-level significance is inflated by the number of cells, these effects are directional support rather than tier anchors. *LXN* was direction-discordant, being RNA-up in bulk blood but down in Jäkel oligodendrocytes. Suggestive *HLA-E* had the largest cell-level effect (Beltrán CD8 T cells, log_2_FC = −1.57, FDR = $5.0\times 10^{-18}$), but by the same rule this is a cell-level rather than a donor-level result and therefore directional support only; *HLA-E* is held at Tier-2 auxiliary rather than promoted to Tier-1. Among additional displayed genes, *ICAM1*, *ITGAL* and *CASP6* retain their respective Tier-2 classifications; *FOXP3*, *TYK2* and *IFI44L* are shown only as immune context.

Because cell-level significance is inflated when cells from the same donor are treated as independent replicates, the candidate panel was re-tested at the level of biological samples in the Kaufmann cohort, the only single-cell cohort with sufficient donor replication (31 matched MS–HC donor pairs; Section 4.7). In the whole-PBMC pseudobulk (11,409 genes tested), *IKZF1* retained significance across the candidate panel (log_2_FC =−0.078, $p=3.6\times 10^{-3}$, BH-FDR =0.047), with the direction matching its bulk-RNA down-call. The same contrast in PBMC T cells illustrates the difference between the two levels: a cell-level BH-FDR of $9.4\times 10^{-71}$ becomes a donor-level *p* = $3.9\times 10^{-4}$, so the direction is preserved while the magnitude of the cell-level significance reflects the number of cells rather than the number of donors. *ITGB2* and the remaining candidates were tested in the same analysis but did not reach donor-level significance, and no candidate reached transcriptome-wide significance (best BH-FDR =0.44); their single-cell evidence is therefore retained as directional support, as stated above. No differences in cell-type proportions between MS and controls were detected at any resolution (all BH-FDR >0.19); a non-significant test does not establish that the proportions are equal, but it provides no evidence that the donor-level results are driven by composition shifts, and a variance-component analysis showed the contrast to be donor-limited: the cell-sampling error of each donor mean is only 8–34% of the between-donor standard deviation, so the effective sample size is the 62 donors rather than the 497,705 cells.

**Figure 6:**
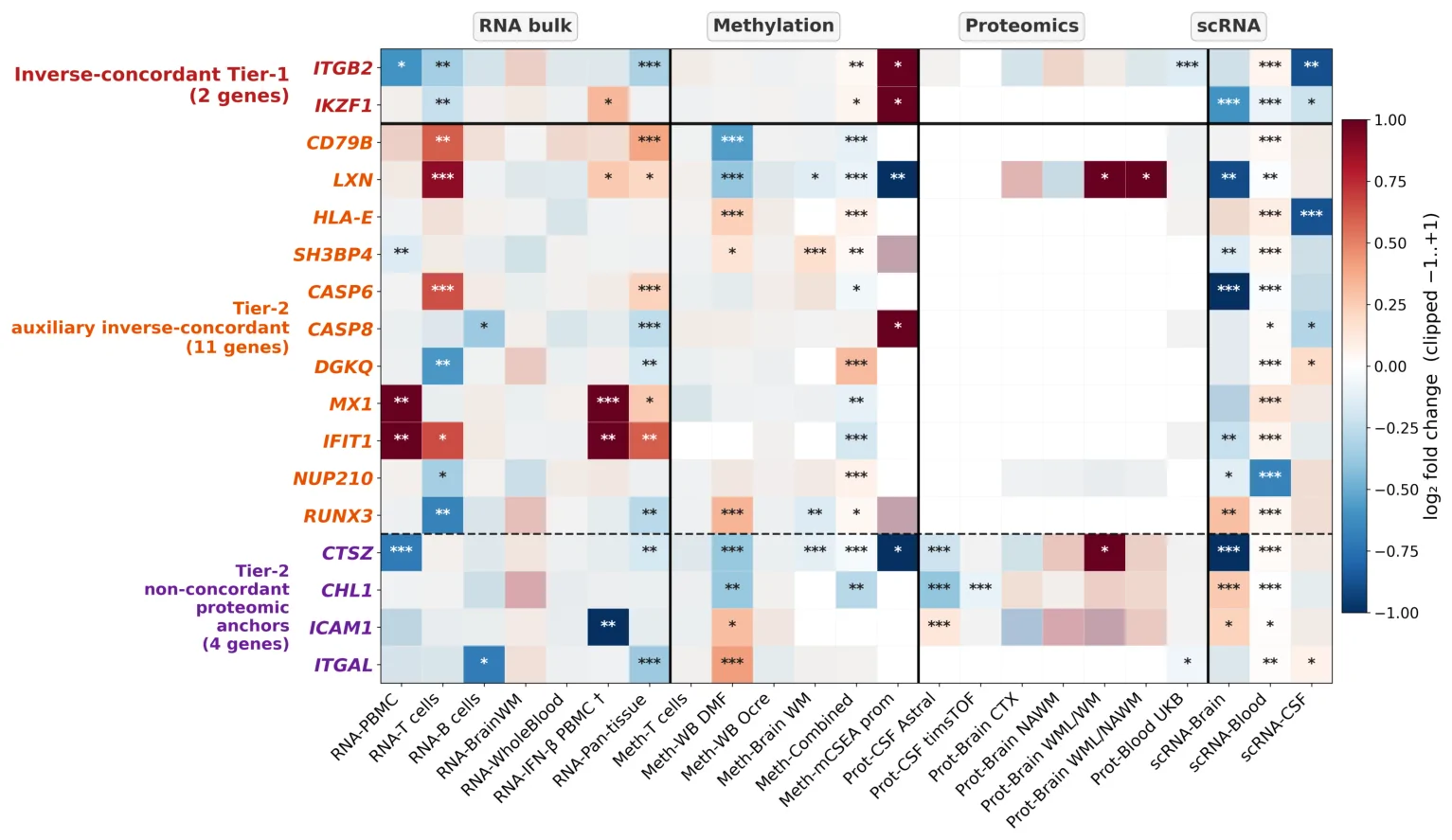
Cross-modal intersection matrix of the 17 tiered candidates across 23 cohort–tissue readouts. Rows comprise three evidence groups: two inverse-concordant Tier-1 genes, eleven Tier-2 auxiliary inverse-concordant genes and four Tier-2 non-concordant proteomic anchors. Columns represent bulk RNA, DNA methylation, proteomics and single-cell readouts. Colour encodes log_2_FC or UK Biobank association β (clipped to ±1); non-significant measurements are faded and white indicates no data. Asterisks denote BH-FDR significance (* BH-FDR < 0.05; ** BH-FDR < 0.01; *** BH-FDR < 0.001), except in the underpowered brain-proteomic contrasts, where they denote nominal *p*-values on the same three levels. The dagger (†) on the column label RNA-IFN-β PBMC marks the one column that is not an MS vs. control contrast: it is the interferon-β treatment-response comparison (IFN-β vs. baseline), which is shown as treatment context only and is excluded from the inverse-concordance scan (Section 4.5). Single-cell columns are cell-level directional comparisons; donor-level pseudobulk determines whether a gene receives a single-cell anchor. The final methylation column, Meth-mCSEA prom, reports the region-level mCSEA promoter test rather than a per-CpG contrast; it is included because it is the only assay in which *CASP8* attains methylation significance, and it also carries the promoter evidence for *ITGB2*, *IKZF1*, *LXN* and *CTSZ*. Its statistic is a directional enrichment score on a wider scale than the β-based columns to its left, so under the shared ±1 clip, its colour saturates, and only its sign and its significance markers should be interpreted, not the intensity of its shading.

Figure 6 consolidates the 17 tiered candidates across 23 RNA, methylation, proteomic and single-cell readouts. The opposing RNA and methylation effects of *ITGB2*, *IKZF1*, *SH3BP4*, *CD79B* and *LXN* make the inverse-concordant rule directly visible, while the proteomic and single-cell columns show the orthogonal evidence bearing on the Tier-1 anchor, a gene reaching Tier-1 only when that evidence includes a significant proteomic and/or donor-level single-cell effect.

### 2.7. STRING Physical-Interaction Network and Pathway Enrichment

**Figure 7:**
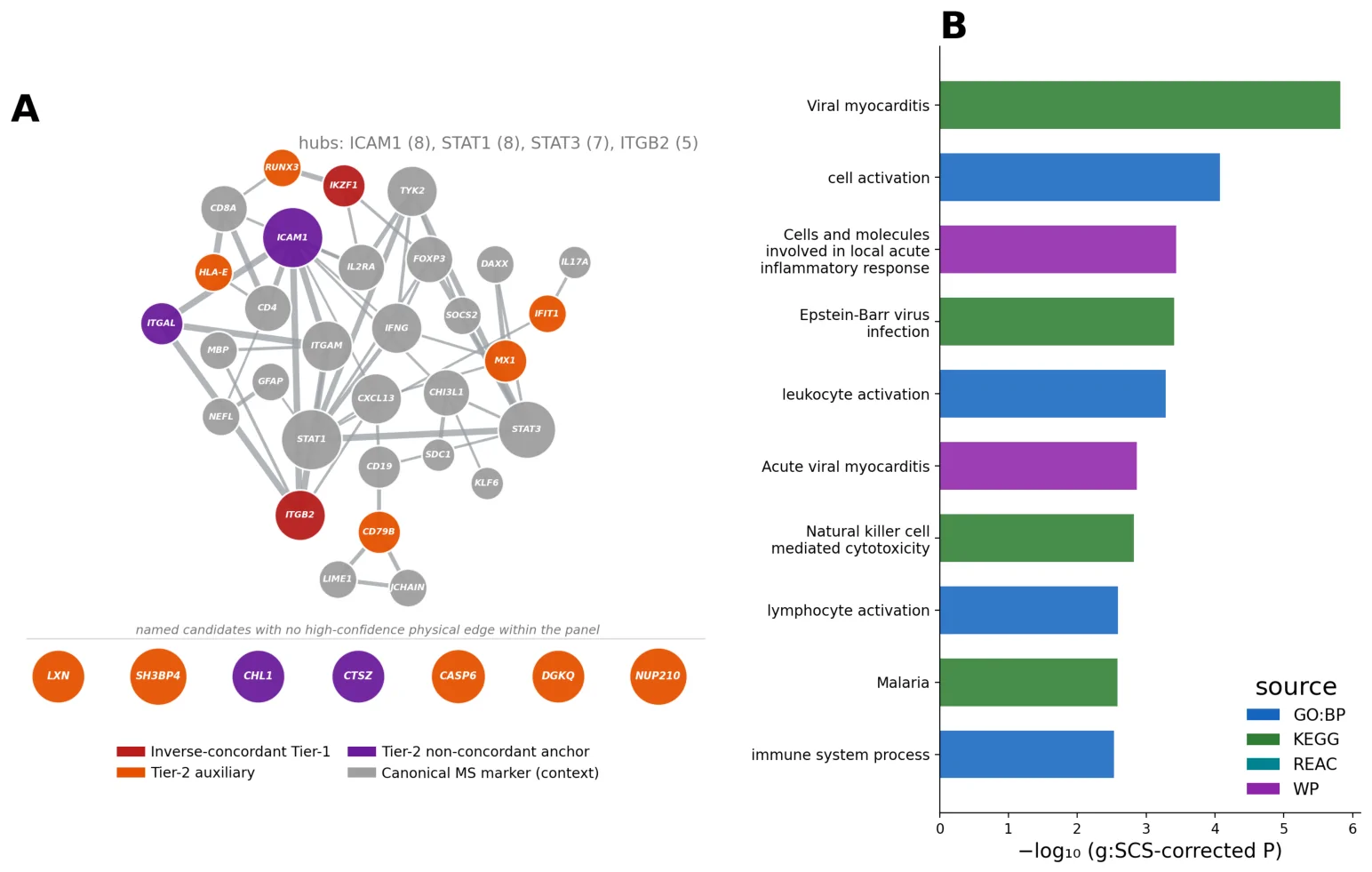
STRING physical-interaction network and pathway enrichment. (**A**) The 38-gene panel contains 17 tiered candidates and 21 canonical MS immune/CNS context genes. Nodes use the common evidence colours; grey is reserved for context. Node size is proportional to within-panel degree and edge width to STRING score. Thirty-one genes form 53 physical edges around the ITGB2–ITGAL–ITGAM–ICAM1 adhesion hub, IKZF1–RUNX3 and STAT1/STAT3 regulatory modules, the CD79B–CD19 B-cell-receptor module, and IFIT1/MX1 interferon wiring. Seven tiered candidates without a high-confidence within-panel edge (*LXN*, *SH3BP4*, *CASP6*, *CTSZ*, *CHL1*, *DGKQ*, *NUP210*) are shown below with enlarged nodes to preserve their labels. (**B**) g:Profiler g:SCS pathway enrichment of the strict 17-gene tiered panel, showing the ten most significant terms. *HLA-E* is included in this enrichment as a Tier-2 auxiliary gene. The KEGG and WikiPathways disease categories among these terms are driven by the leukocyte-adhesion genes *ITGB2*, *ICAM1* and *ITGAL* and should be read as generic immune-adhesion annotation, not as evidence of the named infections.

STRING analysis of the 38-gene display panel (17 tiered candidates and 21 context genes) recovers 31 connected genes and 53 physical edges (Figure 7A). The network centres on an ITGB2–ITGAL–ITGAM–ICAM1 adhesion hub, an IKZF1–RUNX3/FOXP3–STAT1–STAT3 regulatory axis, a CD79B–CD19 B-cell-receptor module and IFIT1/MX1 interferon wiring. Seven tiered candidates (*LXN*, *SH3BP4*, *CASP6*, *CTSZ*, *CHL1*, *DGKQ*, *NUP210*) have no high-confidence within-panel edge. g:Profiler enrichment was restricted to the strict 17-gene tiered panel (Figure 7B), which returns 29 significant terms. The leading terms are immune-cell activation (Gene Ontology [GO] cell activation, $p=8.5\times 10^{-5}$, 9 genes; leukocyte activation, $p=5.2\times 10^{-4}$, 8 genes) together with a group of Kyoto Encyclopedia of Genes and Genomes (KEGG) and WikiPathways disease categories: viral myocarditis, local acute inflammatory response, Epstein–Barr virus infection, malaria, *Staphylococcus aureus* infection and rheumatoid arthritis. Including *HLA-E* in the 17-gene panel also brings in KEGG natural-killer-cell-mediated cytotoxicity ($p=1.5\times 10^{-3}$, 4 genes), the pathway its CD94/NKG2A (natural killer group 2 member A) biology predicts. Those disease categories are carried almost entirely by the same three adhesion genes, *ITGB2*, *ICAM1* and *ITGAL* (joined by *CASP8* in two of them), and therefore reflect shared leukocyte-adhesion annotation rather than pathogen-specific biology; two of them, malaria and *S. aureus* infection, are not viral at all. GO lymphocyte activation is present but weaker (p=0.026) and falls outside the ten terms displayed.

As a check against curated disease catalogues (DisGeNET [27], GWAS Catalog [28], Jensen DISEASES [29]), 25 of the 94 inverse-concordant genes, including two Tier-1 (*ITGB2*, *IKZF1*), the Tier-2 auxiliary genes *SH3BP4* and *HLA-E*, appeared in curated MS/autoimmune gene sets, and against the conservative 8277-gene background, the pool is enriched for autoimmune disease terms (DisGeNET Autoimmune Diseases 19/594, *q* = 0.041; Jensen DISEASES Rheumatoid arthritis 9/168, *q* = 0.015), although the multiple-sclerosis terms themselves remain nominal (DisGeNET Multiple Sclerosis 15/611, *q* = 0.20; GWAS Catalog Multiple Sclerosis 4/140, *q* = 0.22).

## 3. Discussion

Prioritising genes by cross-layer agreement rather than by single-layer candidate lists [5,6,7] yielded a compact mechanistic core. A strict bulk-RNA × methylation inverse-concordant filter across 30 harmonised datasets, followed by independent proteomic and/or donor-level single-cell anchoring, retained two Tier-1 candidates, *ITGB2* and *IKZF1*, and held the remaining inverse-concordant genes at Tier-2 auxiliary. Below, we integrate these axes, position them against prior work and delimit their interpretation.

*ITGB2* encodes the CD18 β2-integrin chain shared by lymphocyte function-associated antigen 1 (LFA-1) and macrophage-1 antigen (Mac-1), which mediate leukocyte arrest and transmigration across inflamed endothelium through ICAM-1 [30,31,32,33,34]. It was the most coherent candidate: RNA down-regulation across blood and brain strata, increased methylation across eight pairings, and independent reduction in UK Biobank MS plasma (β = −0.145, FDR = $1.5\times 10^{-17}$). The broader principle of therapeutically targeting leukocyte trafficking is clinically validated by natalizumab; however, natalizumab blocks α4-integrin rather than ITGB2, whereas efalizumab engaged the ITGAL/ITGB2 LFA-1 heterodimer. We therefore interpret ITGB2 as a proteomically anchored marker of leukocyte trafficking, not as the direct target of an approved MS therapy [35,36,37,38].

The CSF data do not provide a second protein anchor. The complete-case estimate was null (log_2_FC = +0.046, FDR = 0.55), and group-dependent missingness made a left-censored imputed MS-up effect unreliable. Detection, but not abundance among measured samples, tracked CSF leukocyte count, consistent with pleocytosis affecting assay coverage rather than ITGB2 concentration [39]. Migration-coupled shedding of CD11/CD18 complexes is biologically plausible [40,41,42,43], but has not been demonstrated in MS CSF. Resolving this compartmental discrepancy requires targeted ITGB2 measurement in paired plasma/CSF with explicit adjustment for CSF cell count.

*HLA-E* is retained as a Tier-2 auxiliary, hypothesis-generating candidate. Its bulk-RNA and methylation arms are both significant and oppositely directed, but it has no qualifying orthogonal anchor: it is not quantified in either CSF proteome nor in any of the four brain-region contrasts, it is non-significant in UK Biobank plasma (β = +0.037, FDR = 0.14), and it shows no donor-level single-cell difference. Its large CSF CD8 T-cell effect is a cell-level observation, which the tier rule counts as directional support rather than as a qualifying layer; *CD79B* and *LXN* are placed at Tier-2 on the same basis. Its single-cell CSF CD8 T-cell signal and prior MS-lesion findings [44,45] mark a non-classical class-I/NKG2A immune-tolerance axis [46]. Mechanistically, cell-surface HLA-E engages the inhibitory CD94/NKG2A receptor on natural-killer (NK) cells and cytotoxic CD8 T cells, transmitting an immune-checkpoint signal that suppresses degranulation and target-cell killing. Thus, if the *HLA-E* decrease is biologically real, the predicted chain is HLA-E down-regulation → weaker NKG2A-mediated inhibition → disinhibited NK/CD8 cytotoxicity. The NKG2A-blocking antibody monalizumab [47] also releases this inhibitory brake; it would therefore reinforce, rather than reverse, the putative disease-associated functional direction and is not an immediate repurposing candidate for MS.

*IKZF1* encodes Ikaros, a zinc-finger transcription factor that is a master regulator of lymphocyte lineage commitment and development [21], and that registered as inverse-concordant in our bulk RNA × methylation analysis (transcript-down, promoter-hypermethylated). *IKZF1* is not itself a named genome-wide MS GWAS locus; its relevance is grounded instead in the IMSGC enrichment of MS susceptibility signal within peripheral immune-cell and lymphocyte-lineage programmes [4] together with its core developmental biology. Its translational appeal is that Ikaros is the substrate of cereblon-modulating immunomodulatory drugs: cereblon-recruiting agents drive the targeted degradation of IKZF1 and IKZF3 [20], providing a precedented pharmacological handle on a transcription factor that would otherwise be considered undruggable. A regulated reduction in Ikaros-dependent lymphocyte programmes is mechanistically congruent with the broader immunomodulatory rationale in MS and motivates careful, MS-specific evaluation of cereblon-modulator pharmacology rather than direct oncology-dose repurposing.

*CD79B* is the signalling β-chain of the B-cell receptor (BCR) complex and carries the strongest bulk-RNA evidence in the panel (FDR = $8.4\times 10^{-6}$) together with a methylation call whose probe-level support is entirely promoter-derived (three promoter CpGs, all hypomethylated in the same direction, no gene-body contribution); those three probes are too few to constitute a testable mCSEA region, so its promoter support is probe-level only and was never region-tested (Section 2.4). It is nevertheless placed at Tier-2 rather than Tier-1, because *CD79B* has neither of the anchors the Tier-1 criterion requires (Section 2.1): it is not significant in the only proteomic assay in which it is quantified (UK Biobank-PPP plasma, β = −0.052, FDR = 0.20; it is absent from both CSF instruments and from all four brain-region contrasts), and it shows no donor-level single-cell difference. Its single-cell footprint is real but cell-level only (a direction-consistent up-regulation in Kaufmann PBMC monocytes (log_2_FC = +0.18, BH-FDR = $1.2\times 10^{-10}$) and T cells (log_2_FC = +0.04, BH-FDR = $6.6\times 10^{-3}$), and the small per-cell effect sizes mean that significance is driven by cell number rather than by donor-level replication, so it is interpreted as directional support and not as a qualifying layer. As with *IKZF1*, *CD79B* is not an individually named MS GWAS hit; its relevance derives from the IMSGC mapping of MS heritability onto peripheral B-cell and immune-cell pathways [4] and from BCR lineage biology. Its inclusion is biologically timely because B-cell-directed therapy is now firmly established in MS: anti-CD20 depletion with rituximab demonstrated efficacy in relapsing-remitting disease [48], and ocrelizumab subsequently delivered pivotal randomised-controlled evidence across the relapsing and primary-progressive spectrum [49,50]. Consistent with this immunological logic, interferon-β, a first-line MS therapy, down-regulates B-cell-receptor-pathway genes, *CD79B* among them, in patient-derived B cells [51], reversing the transcript-up arm of the inverse-concordant call (*CD79B* is RNA-up in MS). More broadly, intrathecal B-cell activation is an established CSF-biomarker axis in MS [52], and CSF proteomic profiling now resolves protein panels that discriminate MS from controls and track disease activity and disability [26,53,54], the compartment in which a dysregulated BCR component such as *CD79B* would be expected to carry signal. A BCR-complex component that is dysregulated at both the regulatory and expression layers is therefore consonant with the central role of the B-cell compartment in MS pathobiology and offers a candidate marker for follow-up alongside, rather than in place of, depletive therapy.

*LXN* (latexin) is the strongest discovery-stage Tier-2 auxiliary candidate: it carries the strongest methylation evidence in the entire discovery pool (methylation FDR = $1.3\times 10^{-12}$), the lowest mCSEA promoter *q* of any candidate (0.0024), and unanimous probe direction (8 of 8 CpGs hypomethylated), yet it is placed at Tier-2 rather than Tier-1 because it lacks an orthogonal anchor: its proteomic support is nominal only (up in white-matter lesions versus MS-NAWM, log_2_FC =+1.70, p=0.015, but far from surviving correction in this underpowered cohort, FDR =0.97) and its single-cell signal is direction-discordant. It reaches joint RNA × methylation significance in the MS vs. control T-cell and pan-tissue meta-summary strata (with additional treatment-modulation support in the IFN-β PBMC response contrast), with the transcript up-regulated and the promoter hypomethylated in MS blood and T-cell strata. This direction is mechanistically coherent with latexin’s established epigenetic control: latexin, the only endogenous mammalian carboxypeptidase-A inhibitor, is a tumour-suppressor canonically silenced by promoter CpG hypermethylation across human cancers [55,56], so the MS-associated promoter hypomethylation we detect predicts precisely the transcript up-regulation we observe, and the inverse-concordant signature recapitulates a known regulatory axis rather than an arbitrary anticorrelation. Functionally, latexin acts in haematopoietic stem and progenitor cells as a negative regulator of population size, enhancing apoptosis and restraining self-renewal [55,57]; its up-regulation in the MS peripheral immune compartment is therefore consistent with altered lymphocyte homeostasis in autoimmunity, although a direct role in MS immunopathology has not been tested. Two caveats temper the interpretation: latexin’s characterised biology is largely haematopoietic and oncological [55,56,58], with its CNS and oligodendrocyte function essentially uncharted; and the single-cell layer showed a tissue-specific divergence rather than a clean concordant match: *LXN* is transcript-up in blood and T cells yet down-regulated in brain oligodendrocytes (Jäkel Oligo3, log_2_FC = −3.75). *LXN* is therefore best advanced as a blood-compartment biomarker and a mechanistic hypothesis for cell-type-resolved follow-up rather than a unidirectional CNS effector.

*SH3BP4* is placed at Tier-2 auxiliary rather than Tier-1. It has only one inverse-concordant PBMC pairing; promoter evidence is weak (mCSEA q=0.18, 0/11 individual promoter probes significant), with stronger gene-body than promoter support in the DMF stratum; it is not quantified in any proteomic cohort; and neither donor-level pseudobulk nor the direction of its strongest cell-level effect supplies an orthogonal anchor. Its roles in Rag-dependent mTORC1 restraint [59] and transferrin trafficking [60] remain mechanistically relevant, but the present signal supports only an immunometabolic hypothesis requiring promoter-resolved and orthogonal replication.

Read together rather than one at a time, the candidates are not a set of unrelated hits: they sample four coupled stages of a single peripheral process (how the circulating leukocyte pool is specified, polarised, licensed for adhesion, and finally checked), and the physical-interaction network resolves into modules that follow that order. We set this out as an explicit and falsifiable framework, not as an established mechanism. The first stage is lineage specification. The inverse-concordant Tier-1 gene *IKZF1* (transcript-down, promoter-hypermethylated) is a master regulator of lymphocyte lineage commitment [21] and pairs physically with the Tier-2 auxiliary gene *RUNX3* (STRING 0.785), itself a Runx-complex determinant of the CD4/CD8 lineage decision [61], and itself inverse-concordant in the whole-blood meta-cohort; in the B lineage, *CD79B* (transcript-up) anchors a separate B-cell-receptor module with *CD19* [62,63]. The second stage is the cytokine and interferon context in which those programmes are read out: FOXP3 joins the STAT1–STAT3–TYK2 T helper 17 (Th17)/T helper 1 (Th1) cluster, consistent with the STAT3-dependent antagonism between the FOXP3 and Th17 programmes [64], and the inverse-concordant Tier-2 genes *MX1* and *IFIT1* wire into the same cluster through STAT1 and IFNG, placing a type-I interferon axis, the pathway engaged by first-line interferon-β therapy [65], alongside the lineage factors rather than in a compartment of its own. The third stage is adhesion and transendothelial migration, and it is the only stage carrying a population-scale orthogonal anchor: the inverse-concordant Tier-1 gene *ITGB2* sits in a β2-integrin hub with ITGAL, ITGAM and ICAM1 (STRING ≥ 0.99), the axis through which leukocytes engage the blood–brain barrier [32]. ITGB2 is in turn the fourth-most-significant protein in the UK Biobank-PPP MS plasma proteome, and two of the four top-ranked proteins (ITGAV, NEFL, ITGAM, ITGB2) are subunits of that same β2-integrin axis, ITGAM and ITGB2 itself, so the orthogonal signal is not confined to a single protein. The fourth stage is the cytotoxic checkpoint, and here, only the Tier-2 auxiliary gene *HLA-E* contributes: its strongest evidence is the largest cell-level effect in the panel, a down-regulation in CSF and blood CD8 T cells, which under the CD94/NKG2A logic set out above [46,47] would disinhibit rather than restrain effector cytotoxicity. The unifying proposition is therefore that MS-associated inverse-concordant methylation–expression associations in the periphery concentrate in the programmes determining which leukocytes exist, how they are polarised, whether they can cross the blood–brain barrier, and how their killing is checked, and that *ITGB2* is where that chain is currently best anchored, because it is the only element of it with an independent population-scale protein measurement.

Three limits belong with the framework rather than after it. First, our design fixes the direction of coupling but not the direction of causation: an inverse RNA–methylation pairing is equally compatible with methylation constraining transcription, with transcriptional activity reshaping local methylation, and with a shift in the cellular composition of blood generating both, and nothing in the present data adjudicates between them. Second, the framework does not accommodate every candidate. Seven of the named genes (*LXN*, *SH3BP4*, *CASP6*, *CTSZ*, *CHL1*, *DGKQ* and *NUP210*) carry no high-confidence physical edge within the panel, and two of them are precisely the leads with the most distinctive biology: *LXN*, whose blood-up/brain-down divergence is tissue-specific rather than pathway-specific, and *SH3BP4*, an mTORC1- and iron-trafficking lead with no proteomic measurement in any compartment. These sit outside the scheme above and are better advanced on their own terms. Third, the framework is a scaffold for prioritisation and for designing the confirmatory experiments named in this section; the evidential weight of this study rests on the tier assignments and the per-gene cross-layer tables rather than on the framework itself.

These genes also participate in immune-regulatory pathways shared across immune-related diseases, while the direction, consistency, and convergence of their dysregulation across multiple molecular layers in MS tissue further support their specific relevance to MS pathogenesis. *IKZF1* is a genome-wide-significant susceptibility locus for systemic lupus erythematosus (SLE) [66], and the allele that raises childhood B-cell-precursor acute lymphoblastic leukaemia risk protects against type 1 diabetes [67]: antagonistic pleiotropy at one locus, which by itself forbids reading the *IKZF1* signal as disease-specific and instead places it within a shared lymphoid and type-I-interferon autoimmunity programme. The *ITGB2*/CD18 axis also recurs in immune-mediated joint disease: soluble CD18 complexes are released in inflammatory arthritis [41], and reduced plasma soluble CD18 tracks leukocyte infiltration and disease activity in spondyloarthritis [43]. These protein-level observations support a shared leukocyte-trafficking mechanism, but do not establish *ITGB2* as a causal autoimmune susceptibility gene. For *CD79B*, the closest evidence is functional and preclinical: anti-CD79b modulation ameliorated experimental autoimmune encephalomyelitis, the principal mouse model of MS [62], and suppressed collagen-induced arthritis more effectively than anti-CD20 depletion [63]. These experiments make BCR signalling through CD79B relevant across neuroinflammatory and systemic autoimmunity, while remaining target-validation studies rather than human genetic associations. *HLA-E* likewise connects to autoimmune tolerance beyond MS: HLA-E-restricted regulatory CD8 T-cell dysfunction was demonstrated in human type 1 diabetes [68], and *HLA-E* polymorphisms were associated with rheumatoid-arthritis susceptibility and anti-tumour necrosis factor (TNF) response in a single female cohort [69]. Together, these findings position the HLA-E–NKG2A checkpoint as a shared tolerance pathway, although neither the cohort-specific genetic signal nor the functional type 1 diabetes result proves an MS-specific role.

The non-autoimmune associations reinforce the same specificity caution. *IKZF1* is also a childhood B-cell-precursor acute lymphoblastic leukaemia locus [70]; germline heterozygous variants cause combined immunodeficiency [71], somatic deletion is an adverse prognostic lesion in paediatric leukaemia [72], and IKZF1 is the degradation substrate through which lenalidomide acts [73]. *ITGB2* has a narrow Mendelian footprint (biallelic loss causes leukocyte adhesion deficiency type I [74,75]), and in the GWAS Catalog [28] (queried on 4 August 2026 for variants mapped to *ITGB2*; 36 of 47 associations at $p<5\times 10^{-8}$), every genome-wide-significant association is a molecular or quantitative trait rather than a disease diagnosis, chiefly protein quantitative trait loci for ITGB2 and neighbouring integrins, surface CD11b intensity, lymphocyte count and bone mineral density, while the reported disease-level candidate single-nucleotide polymorphism (SNP) associations are small and unreplicated [76]; at the expression and protein level, it recurs as a network hub in vascular dementia [77] and as a nominated causal plasma protein in lung adenocarcinoma [78], the latter derived from the same UK Biobank plasma resource that anchors our own result. *CD79B* additionally causes autosomal-recessive agammaglobulinaemia [79], carries somatic mutations that drive chronic active B-cell-receptor signalling in diffuse large B-cell lymphoma [80], and is targeted by polatuzumab vedotin [81]. *LXN* is known mainly as a haematopoietic stem-cell quantitative-trait gene [57] and a promoter-hypermethylated candidate tumour suppressor [56,58]; the nearest inflammatory evidence is a macrophage-focused atherosclerosis study in which *LXN* deletion promoted an anti-inflammatory phenotype and reduced plaque burden in mice [82], not a human autoimmune association. For *SH3BP4*, no replicated human autoimmune-disease association has been reported, so its relevance remains pathway-level through mTORC1 and iron trafficking. We therefore position the candidates as readouts of shared lymphoid specification, B-cell signalling, leukocyte adhesion and immune-tolerance programmes whose MS relevance rests on the direction and cross-layer coherence of their change in MS tissue, not on any claim that the genes are MS-specific.

Underlying the prioritisation is a regulatory premise that warrants explicit framing as a concept rather than an established MS-specific mechanism. Three caveats qualify the regulatory rationale set out in the Introduction. The methylation–transcription relationship is context-dependent, and intragenic methylation can accompany active transcription rather than silencing [16]. The precedent for reading directional discordance as a disease signal comes from leukaemia rather than MS [17], so the filter is a framing device for candidate selection and not a validated MS regulatory mechanism. What it buys is a narrower candidate list rather than freedom from confounding: same-direction changes are the ones most readily explained by cellular-composition shifts and shared cytokine programmes, so requiring inverse concordance down-weights that particular class of signal while leaving intact the possibility noted above that a composition shift generates the inverse pairing itself.

Positioned against prior MS multi-omics work, this study occupies a distinct niche. Population-genetic integrations anchored on the IMSGC reference map have nominated susceptibility genes by colocalising GWAS signal with expression quantitative trait locus (eQTL) catalogues and have established that MS heritability is enriched in peripheral immune cells, monocytes and microglia [4], but they do not interrogate methylation or proteomic layers and historically lacked single-cell resolution. Epigenome-wide association studies have assembled large MS methylation cohorts and identified cell-type and *HLA-DRB1*-region signals [6], and sorted CD4+/CD8+ T-cell methylation studies have begun to resolve cell-type-specific marks [83,84,85,86], yet these typically lack matched transcriptomic or proteomic anchoring. Single-layer transcriptomic [5] and CSF proteomic [7,53] studies likewise generate candidates without cross-layer corroboration. By requiring strict bulk RNA × methylation inverse concordance and then demanding an independent proteomic and/or donor-level single-cell anchor, our pipeline trades breadth for a smaller, more conservative candidate set. Within the overlap with peripheral-immune MS biology, *ITGB2* stands out as proteomically replicated, whereas *LXN*, *CD79B* and *SH3BP4* are prioritisations that GWAS-only approaches would not have surfaced.

Relevant comparator studies used substantially different study designs. Elkjaer et al. [87] systematically reviewed MS brain transcriptomic and proteomic studies but did not integrate statistics or prioritise genes; their lesion atlas [88] integrated single-nucleus RNA-seq, assay for transposase-accessible chromatin using sequencing (ATAC-seq) and spatial transcriptomics in progressive-MS brain, without methylation or proteomics. Xavier et al. [89] and Ewing et al. [90] focused on DNA methylation, using large deconvolved whole-blood and purified immune-cell designs, respectively.

Our strongest agreement with the methylation studies is compartmental. Both prioritised B-cell and monocyte signals; in our orthogonal sorted-cell cohort, significant candidate promoters were likewise concentrated in B cells (*RUNX3*, *CTSZ*, *ICAM1*), the sole signal outside that compartment being *ICAM1* in CD4 T cells (BH-FDR = 0.046), with nominal direction-consistent *CD79B* hypomethylation in B cells. This is not formal replication: our sorted arms are smaller, and *RUNX3* direction differs between the two prior studies. The comparators are also stronger in specific respects: Xavier et al. included genotypes, methylation quantitative trait locus (methQTL) and mediation analyses; Ewing et al. used purified cells and disease-stage contrasts; and Elkjaer et al. measured modalities in the same lesions. Our layers instead come from different cohorts joined at the gene level, and genotype independence cannot be established.

The present design adds two orthogonal filters absent from those studies: population/CSF/brain proteomics and donor-level single-cell validation. Their combination restricts Tier-1 to *ITGB2* and *IKZF1*, holding *CD79B*, *LXN* and *SH3BP4* at Tier-2 despite strong single-layer signals. The inverse-concordant methylation–expression direction itself is not new: Huynh et al. [91] demonstrated it in MS brain, and GSE40360 from that work is re-analysed here. The contribution is its uniform use as a cross-stratum screen followed by an independent anchor. These remain cohort-level associations rather than within-donor coupling, and most methylation calls are promoter-plus-gene-body composites.

Two safeguards materially shape the interpretation: disease status was protected during batch correction, and single-cell tier credit required donor-level rather than cell-level replication. Further details of the statistical controls are provided in Section 4.

Several limitations bound these findings. The public cohorts are predominantly of European ancestry, and discovery and validation layers contain different individuals; cross-layer agreement is therefore biological replication, not within-person concordance. Sorted-cell methylation arms are small (approximately 10–26 samples per group), and neither Tier-1 promoter was significant in an individual sorted population. Two methylation series lacking IDATs (GSE106648 and GSE40360) contribute 326/475 samples and could not receive the raw detection-p filter, so residual preprocessing heterogeneity remains possible despite satisfactory ComBat diagnostics. GSE106648 also overlaps the validation data used by Xavier and Ewing [6,89,90], limiting independence of that comparison. Sex was unresolved for 66 transcriptomic samples; sex-adjusted sensitivity analyses were stable, but age was unavailable for most expression cohorts and residual age effects cannot be excluded, particularly in GSE40360. Treatment strata are small, making the *HLA-E* and *LXN* treatment-context signals preliminary. Finally, the human leukocyte antigen (HLA) region is complex, and the data do not assign the *HLA-E* phenotype to a causal MHC variant.

Two extensions follow directly. First, long-read RNA sequencing would test whether the inverse-concordant methylation–expression association we observe at the gene level is partly driven by isoform-switching that short-read bulk sequencing cannot resolve. Second, paired plasma proteomics from the same subjects as the CSF cohorts would establish whether the ITGB2 plasma signal and the *LXN*/*CD79B*/*SH3BP4* leads generalise to a less invasive sampling matrix.

## 4. Materials and Methods

### 4.1. Data Sources and Cohort Assembly

The integrated multi-omics atlas comprises 30 publicly deposited primary datasets across four molecular layers (15 bulk-transcriptomic series, 9 DNA-methylation series [eight arrays plus the GSE173787 whole-genome bisulfite-sequencing set], 3 single-cell cohorts and 3 deposited proteomic datasets [the Bader CSF Astral, Bader CSF timsTOF and Wang & Julien brain proteomes]; the UK Biobank-PPP plasma summary statistics and the curated 30-study proteomic meta-cohort are integrated as additional external validation and are not counted among the 30; per-study annotation with verified GSE/PRIDE (PRoteomics IDEntifications database) accessions and PubMed identifier (PMID) anchors is provided in Supplementary Table S1; sample counts follow the GEO/PRIDE depositor records): (i) Bulk transcriptomics: 15 GEO series totalling 552 samples (367 MS/185 HC), which are the samples entering batch correction and per-stratum testing; GSE66573 (whole blood) contributes 14 samples to the harmonised 727-sample expression matrix but does not enter the 552-sample analysis set or any per-stratum result; the collection covers whole blood, PBMC, neutrophils, purified T and B lymphocytes, and brain cortex/white-matter tissue; key series include GSE103005, GSE21942 (PBMC), GSE288904 (neutrophils), GSE138064 (PBMC [65]), GSE173789 (B cells [92]), GSE190847 (B cells [93]), GSE211358 [94], GSE207680 (cortex [95]), GSE38010 [96] and GSE43591 [97]. (ii) DNA methylation: 8 GEO series on Illumina 450K and EPIC arrays, including the whole-blood cohort (GSE106648; *n* = 140 MS/139 HC [6]), purified T cells (GSE130029 and GSE130030 [83]), CD4 T cells and monocytes (GSE189255 and GSE189256 [98]), the DMF + ocrelizumab whole-blood treatment cohort (GSE219293; *n* = 29 MS/18 HC, EPIC [99]), a whole-blood cohort (GSE88824), and brain white matter (GSE40360 [91]); a further sequencing-based methylation series provides an orthogonal cell-type-resolved assessment: sorted-immune whole-genome bisulfite sequencing (GSE173787; CD19 B cells, CD4 T, CD8 T and CD14 monocytes; 72 MS/57 HC post-quality-control (QC) donor × cell-type profiles (129 of 133 deposited) from 53 donors (29 MS/24 HC) [92]). (iii) Single-cell RNA-seq: three primary cohorts annotated according to their depositor records: brain snRNA-seq (GSE118257; 9 donors, 4 MS/5 HC, across 20 white-matter region samples [11]), PBMC scRNA-seq + CITE-seq (10× Chromium 3^′^; GSE144744, 497,705 cells, 62 donors (31 MS/31 HC) [13]), and MS-discordant monozygotic twin cohort CSF + PBMC (GSE127969; 10 donors in 5 MS-discordant monozygotic twin pairs; 2029 post-QC cells, 1166 from MS twins versus 863 from their co-twin controls, from the deposited MS-discordant twin-pair cohort [14]). (iv) Proteomics: primary cohorts are the Bader & Mann CSF Orbitrap Astral DIA-MS discovery dataset [8] (PRIDE PXD064570) and the Bader & Mann CSF timsTOF dataset (PRIDE PXD045058; the larger ~5000-sample workflow spanning MS, other neurological diseases and healthy controls), together with the Wang & Julien region-resolved brain white-matter proteome [9] (raw spectra on MassIVE, MSV000096790; processed proteomics on Figshare); we additionally integrate the published UK Biobank-PPP Olink plasma proteomic associations (Jacobs et al. [25]; 407 prevalent MS vs. 39,979 controls drawn from 52,705 participants with proteomic data) and a curated meta-cohort of 30 published MS case-control serum/plasma/CSF proteomics studies (173 differentially-abundant proteins; see Supplementary Table S1). Two further MS single-cell cohorts, GSE138266 (MS vs. non-inflammatory neurological disease [12]) and the integrated CSF atlas (Zenodo 6910635 [100]), met the inclusion criteria but were not included in the present analysis.

#### Cohort Inclusion and Exclusion Criteria

Eligible cohorts contained human primary samples, sample-level MS/control labels, at least three biological replicates per arm (except two tissue-rare brain cohorts), and blood, purified immune-cell, CSF or brain tissue. Treatment-experienced arms were analysed separately from untreated case-control strata. Layer-specific quality control excluded unstable bulk-RNA series, methylation samples with >5% failed probes and low-quality/doublet single cells; proteomic tests required ≥50% quantification per group and used observed intensities without imputation. Candidate proteins below that completeness threshold were examined only in an exploratory raw-matrix Mann–Whitney analysis and could not provide a tier anchor.

### 4.2. Sex and Age Composition, and the Treatment of Sex in the Models

Sex and age were taken from deposited Simple Omnibus Format in Text (SOFT) records and phenotype tables. Where sex was absent, it was inferred within cohort from *XIST* and Y-linked expression markers, or from chrX methylation in GSE88824; expression-based calls agreed with all 159 deposited labels available for validation. Sex was resolved for 486/552 bulk-transcriptomic samples, 475/475 methylation samples and all 81 single-cell donors; the two unresolved transcriptomic series are reported as unknown in Supplementary Table S3. Age was available for 4/15 transcriptomic series and 419/475 methylation samples.

The methylation cohort was sex-balanced (67.6% female in MS vs. 69.3% in controls, *p* = 0.78), whereas the resolved transcriptomic cohort was not (72.1% vs. 56.4% female, χ^2^ = 11.1, *p* = $8.5\times 10^{-4}$). Age varied principally by tissue; GSE40360 was the least balanced stratum (MS: 60.7% female, median 54 years; controls: 36.8%, median 68 years). Primary bulk models included condition and, where applicable, dataset/batch but not sex or age; sex-chromosome probes were removed from the methylation analysis. The Kaufmann donor-level single-cell model used pair + condition across 31 sex-matched, age-balanced MS–control pairs.

Sensitivity models added sex on identical sample sets. The transcriptomic cohort is sex-imbalanced (Section 4.2), which is why the sensitivity model was fitted. Disease-effect estimates remained highly concordant in methylation (Pearson *r* = 0.9999 across 270,123 probes) and transcriptomics (*r* = 0.975 across 486 samples), with no sign change among the named candidates and no change in tier assignment. The primary models were therefore retained. Age could not be assessed comparably because it was unavailable for most transcriptomic cohorts.

### 4.3. Bulk Transcriptomic Processing

The bulk transcriptomic datasets comprise two data types, microarray and RNA sequencing, whose feature identifiers differ fundamentally, and were harmonised on separate tracks before integration. (i) Microarray track (six series): per-platform probe identifiers were mapped to HUGO Gene Nomenclature Committee (HGNC) gene symbols using the manufacturer platform annotations (Affymetrix HG-U133 Plus 2.0 for GSE21942, GSE43591 and GSE38010; Illumina HumanHT-12 v4 for GSE103005; Affymetrix HTA-2.0/Clariom-D for GSE138064 and GSE190847). (ii) RNA-seq track (nine series): deposited gene-level quantifications were mapped to HGNC symbols from their Ensembl or RefSeq identifiers, or used directly where symbols were provided. Within each series, probes or transcripts mapping to the same symbol were collapsed by the maximum, and MS vs. control status was taken from the sample metadata. The two tracks were merged on gene symbols present in at least 50% of series, giving a harmonised matrix of 25,460 genes across 727 expression samples. Restriction to genes detected in at least 75% of contributing datasets left 8860 genes across 552 samples (367 MS/185 HC) as the matrix entering batch correction and per-stratum testing. GSE137143 contributes the sorted CD4 and CD8 fractions of 26 donors to the T-cell stratum; because the two fractions of a donor are not independent and its control arm comprises both fractions of a single donor, this series is retained with dataset modelled as a batch covariate and is not treated as an independent 52-sample contrast. Per-dataset scale normalisation (counts-per-million then log_2_ for sequencing data, quantile normalisation for arrays) preceded ComBat [22,101] batch correction (sva v3.60 [101]) with the MS vs. control covariate preserved in the design matrix. Differential expression was computed per tissue/treatment stratum with the limma-trend approach (limma’s linear model with empirical-Bayes moderation accommodating the gene-level mean–variance trend (lmFit followed by eBayes with trend = TRUE and robust = TRUE [23]), at a Benjamini–Hochberg FDR of 0.05. Treatment-experienced arms (the interferon-β time-course in GSE138064, and the dimethyl fumarate and ocrelizumab series) were analysed as their own strata and never pooled with the treatment-naïve MS vs. control discovery set (552 samples).

### 4.4. DNA Methylation Processing

Six array series with IDATs (149 samples) were processed with minfi v1.58 preprocessIllumina [102]; depositor-processed signal matrices were used for GSE106648 (279 samples) and GSE40360 (47 samples), the two series without IDATs, and quantile-normalised within series. (normalize_beta_only.R offers BMIQ as an alternative when wateRmelon is installed; it was not, so the quantile path is what ran.) All series underwent missingness filtering and removal of SNP-affected, cross-reactive and sex-chromosome probes; detection-p filtering (p<0.01) was additionally applied where IDATs were available. The eight series were merged on 270,123 probes, converted to M-values and ComBat-corrected with disease status protected [22]. Per-stratum DMPs used limma, and promoter/gene-body enrichment used mCSEA v1.32 [24]. For an orthogonal cell-type-resolved assessment, GSE173787 sorted-cell whole-genome bisulfite data were aggregated over transcription start site (TSS) ±2 kb promoter windows (GENCODE v49 [103]) and tested per cell type with Welch tests and BH correction.

### 4.5. Inverse-Concordant Cross-Omics Scan

For each gene, inverse concordance required BH-FDR < 0.05 in bulk RNA and methylation from the same tissue stratum, with opposite effects. Only MS-versus-control strata enter this scan. The IFN-β PBMC stratum is a treatment-response contrast and is excluded from it, so that a drug effect cannot define a disease candidate; it is reported as treatment context only (Figure 2C). Seven genes entered the pool through that pairing alone and leave with it; no tiered candidate does. CpG-level methylation *p*-values were combined by an unweighted signed Stouffer statistic, $Z=\sum_{i}\mathrm{sign}\left(b_{i}\right)\Phi^{-1}\left(1-p_{i}/2\right)/\sqrt{n}$; the displayed gene-level effect is the arithmetic mean probe logFC. Precision-weighted, inverse-variance-weighted and probe-range summaries are provided in Supplementary Table S2.

Because the aggregate includes promoter and gene-body probes, it was not interpreted automatically as promoter silencing. Each of the 94 genes was classified as promoter-anchored by mCSEA [24] or supported only by the promoter-plus-gene-body composite, and the analysis was repeated using promoter-significant genes alone (Section 2.4; Supplementary Table S2).

Tier-1 required bulk-RNA × methylation inverse concordance plus an independent proteomic and/or donor-level single-cell anchor, that is, agreement of at least three of the four layers (Section 2.1). Only *ITGB2* (UK Biobank plasma) and *IKZF1* (whole-PBMC pseudobulk) satisfied this rule. Inverse-concordant genes without such an anchor, including *CD79B*, *LXN* and *SH3BP4*, were assigned to Tier-2 auxiliary; strong proteomic candidates lacking a qualifying RNA–methylation pair formed the four-member Tier-2 non-concordant proteomic anchor group (*CTSZ*, *CHL1*, *ICAM1*, *ITGAL*). *HLA-E* is Tier-2 auxiliary on exactly the basis the rule specifies: both of its discovery arms are significant, and it lacks a qualifying proteomic or donor-level single-cell anchor.

### 4.6. Proteomic Processing

The re-analysed CSF and brain intensity matrices were log_2_-transformed, median-normalised and tested with DEP [104] (R package version 1.7.1; the package was removed from Bioconductor at release 3.23 and was therefore installed from source), including MS-run batch where available [23,105]. Two departures from DEP’s documented workflow are deliberate. Missing intensities are not imputed, so DEP::impute is not called; DEP::test_diff accepts the missing values directly. Its adjusted *p*-values are replaced with Benjamini–Hochberg ones, because test_diff hard-codes fdrtool with no argument to change it, and every other layer of this study reports BH-FDR; on the CSF Astral cohort, fdrtool calls 35 proteins against 955 under BH. The equivalence of the two adjustments at the level of effect estimates, and the two DEP behaviours that must be worked around on already-log-scaled input, are documented in dep_bh_equivalence_check.R. Proteins required ≥50% quantification per group and at least two observed values per arm. Missing intensities were not imputed because group-dependent detection can convert left-censored imputation into an apparent abundance effect; all tests therefore used observed values only. Cross-platform agreement between the Bader Astral and timsTOF CSF cohorts was assessed on 1762 shared proteins (Pearson *r* = 0.45). Brain-region effects are reported at nominal p because no protein survived BH correction at *n* = 5–8 per group. UK Biobank-PPP Olink associations (407 MS, 39,979 controls; 2911 proteins) were used as published [25].

### 4.7. Single-Cell Processing

Deposited quality-control and cell-type annotations were retained, and each cohort was reprocessed independently with Scanpy v1.11 [106]: log-normalisation, 3000 highly variable genes, principal-component analysis (50 components for the brain cohort, 40 for the CSF/PBMC twin cohort), batch-balanced *k*-nearest-neighbour integration [107] and Leiden clustering [108]. The third cohort was not re-integrated: its deposited cluster assignments were used as published. Native cohort cell-type labels were preserved. Candidate-gene MS vs. control effects were estimated within cell type by tie-corrected Wilcoxon testing, requiring ≥20 cells per arm and correcting the joint gene × cohort × cell-type matrix by Benjamini–Hochberg.

Donor-level validation used the Kaufmann cohort (31 matched MS–control pairs). Counts were reconstructed from the deposited log-normalised matrix as round($\left(e^{x}-1\right)$× *s*/10,000), with *s* the per-cell total recorded by the depositors; the reconstruction is exact wherever the deposited scale factor is 10,000 and is otherwise approximate, so the aggregated values are treated as counts for the purpose of library-size weighting only. They were aggregated per donor at whole-PBMC, eight-lineage and 25-subset resolutions, requiring at least ten cells per donor–cell-type unit. Summed counts were analysed by edgeR/limma-voom and mean per-cell counts per 10,000 (CP10K) by limma-trend, using pair + condition and protein-coding genes passing expression filters. Sums of per-cell-normalised values were excluded because they reintroduce cell number as an offset. FDR was reported within cell type, across all gene–cell-type tests and across the 25-gene testing panel defined below. Cell-type proportions were tested separately with the same paired design after arcsine-square-root transformation.

### 4.8. Network and Pathway Analysis

The 17 tiered candidates (2 Tier-1, 11 Tier-2 auxiliary inverse-concordant and 4 Tier-2 non-concordant proteomic anchors) were analysed with STRING v12 [18]. For visual context, 21 connected canonical MS immune/CNS genes were added to the 38-gene network display; they were not included in the candidate enrichment. g:Profiler [19] tested the strict 17-gene panel across GO Biological Process, KEGG, Reactome and WikiPathways using g:SCS correction [109] at FDR < 0.05. Disease-catalogue overlap of the full 94-gene discovery pool used a hypergeometric test against the 8277 genes testable in both discovery layers, that is the union of the per-stratum RNA gene universes intersected with the methylation gene universe (Supplementary Table S2). The test was run offline with gseapy v1.1.13 against the Enrichr libraries DisGeNET, GWAS_Catalog_2023 and Jensen_DISEASES, with that background passed explicitly rather than Enrichr’s default whole-genome background.

### 4.9. Quality Control and Statistical Considerations

All discovery cohorts passed layer-specific quality-control procedures rather than a single common gate: within the methylation layer alone, six series were reprocessed from raw IDATs and two from deposited signal or beta matrices, and the single-cell cohorts entered at the quality control deposited by each source consortium. In every layer, the MS vs. control covariate was preserved in the batch-correction design so that disease signal was not removed alongside technical variation. In the bulk transcriptomic layer, genes missing in more than half of a series were dropped, values were per-dataset scale-normalised, and series that could not support a stable batch-correction design, single-condition series and very small batches, were excluded before ComBat (Section 4.3). DNA-methylation series were uniformly reprocessed and ComBat batch-corrected on the 270,123 common 450K probes (Section 4.4); proteomic cohorts retained protein groups quantified in at least 50% of samples per group (DEP default) before differential testing (Section 4.6); and single-cell cohorts entered at the per-cell quality control and cell-type annotation deposited by each source consortium, in which low-complexity and high-mitochondrial-content cells and predicted doublets had already been removed (Section 4.7).

FDR control was performed within the layer using the Benjamini–Hochberg procedure. For the single-cell layer, the effect size was the log_2_FC (MS vs. control) on log-normalised expression, computed with scanpy rank_genes_groups using the tie-corrected Wilcoxon rank-sum test applied per cell type across all cells of a given cell type pooled across donors, retaining cell types with ≥20 MS and ≥20 HC cells and discarding gene × cell-type combinations in which the gene was detected in no cell, with Benjamini–Hochberg correction across the 783 gene × cohort × cell-type comparisons across the 25-gene testing panel (reported in Section 2.6 and Figure 5B). The 25-gene testing panel is the fixed candidate list carried forward into the single-cell layer; the 17 candidates displayed in the main figures are the visualised subset of that panel, and the full panel membership is given in Section 2.6.

### 4.10. Software

All analyses were performed in R 4.6.0 and Python 3.11.15 (scanpy v1.11.5 for single-cell processing); figure rendering used matplotlib 3.10 [110]. Stochastic clustering and integration steps used fixed random seeds.

## 5. Conclusions

We have assembled an MS multi-omics resource that brings together four distinct molecular layers across 30 publicly deposited datasets, and we have demonstrated that the strict bulk-RNA × methylation inverse-concordant filter, when anchored by proteomic and/or donor-level single-cell evidence, prioritises a compact two-gene Tier-1 set (*ITGB2*, *IKZF1*) that is biologically interpretable. *ITGB2* carries the broadest cross-layer support, extending the leukocyte-integrin transmigration axis (parallel to natalizumab’s α4-axis and the withdrawn anti-LFA-1 efalizumab) and orthogonally supported by lower plasma abundance in UK Biobank MS (β = −0.145, FDR = $1.5\times 10^{-17}$); *IKZF1* links GWAS biology to existing cereblon-modulator immunomodulatory drugs through its donor-level single-cell anchor. Below the Tier-1 pair, the resource retains two disjoint follow-up groups. The eleven Tier-2 auxiliary inverse-concordant genes (*CD79B*, *LXN*, *HLA-E*, *SH3BP4*, *CASP6*, *CASP8*, *DGKQ*, *MX1*, *IFIT1*, *NUP210* and *RUNX3*) satisfy both discovery arms but carry no qualifying orthogonal anchor; the first three named hold the strongest single-layer evidence in the panel and are the most immediate biomarker and mechanistic targets for cell-type-specific follow-up. The four Tier-2 non-concordant proteomic anchors (*CTSZ*, *CHL1*, *ICAM1* and *ITGAL*) carry strong protein-level evidence without a qualifying RNA–methylation pair, and are retained as compartment-specific leads rather than as inverse-concordant candidates. The wider value of the work lies beyond this candidate list: The pipeline, the Tier-1 list and the network are available for any candidate, and the tiering logic–discovery by regulatory–expression discordance, promotion only on orthogonal evidence–generalises to any disease in which such discordance is expected.

## Data Availability

All primary public accessions used in this study are catalogued per-cohort in Supplementary Table S1. NCBI GEO: Bulk transcriptomics (15 series): GSE21942, GSE38010, GSE43591, GSE103005, GSE137143, GSE138064, GSE172009, GSE173789, GSE190847, GSE207680, GSE209596, GSE211358, GSE211739, GSE214334 and GSE288904; GSE66573 contributes to the harmonised expression matrix but not to the 552-sample differential analysis set; DNA methylation (eight Illumina 450K/EPIC array series (GSE40360, GSE88824, GSE106648, GSE130029, GSE130030, GSE189255, GSE189256 and GSE219293) plus the GSE173787 whole-genome bisulfite-sequencing series); single-cell RNA-sequencing (3 series): GSE118257 (Jäkel et al., 2019 [11] brain snRNA-seq), GSE127969 (Beltrán et al., 2019 [14] CSF/PBMC twin pairs) and GSE144744 (Kaufmann et al., 2021 [13] PBMC 10× Chromium 3^′^ scRNA-seq/CITE-seq). PRIDE, proteomics: PXD064570 (Bader & Mann 2026 [8] CSF Orbitrap Astral DIA-MS) and PXD045058 (the same study’s timsTOF CSF dataset); the Wang & Julien 2026 [9] region-resolved brain white-matter proteome is deposited on MassIVE (MSV000096790, raw spectra) and Figshare (processed data). UK Biobank-PPP plasma proteomic associations were taken from the published summary statistics of Jacobs et al., 2024 [25] (Olink Explore; PMID 38282238. doi:10.1002/acn3.51990); individual-level UK Biobank data are available to approved researchers via the UK Biobank Access Management System. All analysis code supporting this study, covering the harmonisation, per-stratum differential testing, inverse-concordance scan, proteomic, single-cell, network and figure-generation steps, is available at https://github.com/alperbulbul1/MS_Omics_Data_Analysis (accessed on 5 August 2026).

## Supplementary Materials

The following supporting information can be downloaded at: https://www.mdpi.com/article/10.3390/ijms27167275/s1.

## Abbreviations

450K	Illumina Infinium HumanMethylation450 BeadChip
API	Application Programming Interface
BCR	B-Cell Receptor
BH	Benjamini–Hochberg (false-discovery-rate procedure)
CD	Cluster of Differentiation
CNS	Central Nervous System
ComBat	Combatting Batch-effects algorithm
CSF	Cerebrospinal Fluid
DAP	Differentially Abundant Protein
DEG	Differentially Expressed Gene
DEP	Differential Enrichment of Proteins (R/Bioconductor package)
DIA	Data-Independent Acquisition
DIA-PASEF	Data-Independent Acquisition with Parallel Accumulation–Serial Fragmentation
DMF	Dimethyl Fumarate
DMP	Differentially Methylated Position
DNA	Deoxyribonucleic Acid
EPIC	Illumina Infinium MethylationEPIC BeadChip
EWAS	Epigenome-Wide Association Study
FC	Fold Change
FDR	False Discovery Rate
GENCODE	GENCODE reference gene annotation
GEO	Gene Expression Omnibus
GO:BP	Gene Ontology Biological Process
GWAS	Genome-Wide Association Study
HC	Healthy Control
HLA	Human Leukocyte Antigen
IDAT	Illumina iScan raw data file
IFN	Interferon
IMSGC	International Multiple Sclerosis Genetics Consortium
KEGG	Kyoto Encyclopedia of Genes and Genomes
LFA-1	Lymphocyte Function-associated Antigen 1
LFQ	Label-Free Quantification
limma	Linear Models for Microarray data
mCSEA	methylated CpG Site Enrichment Analysis
MHC	Major Histocompatibility Complex
MS	Multiple Sclerosis; in DIA-MS, Mass Spectrometry
NAWM	Normal-Appearing White Matter
NES	Normalised Enrichment Score
NK	Natural Killer (cell)
NKG2A	Natural Killer Group 2 member A
OND	Other Neurological Diseases
OPC	Oligodendrocyte Progenitor Cell
PBMC	Peripheral-Blood Mononuclear Cells
PCA	Principal Component Analysis
PML	Progressive Multifocal Leukoencephalopathy
PPI	Protein–Protein Interaction
PPP	Pharma Proteomics Project (UK Biobank)
PRIDE	PRoteomics IDEntifications database
QC	Quality Control
REAC	Reactome
RNA	Ribonucleic Acid
scRNA-seq	Single-cell RNA sequencing
SLE	Systemic Lupus Erythematosus
snRNA-seq	Single-nucleus RNA sequencing
STRING	Search Tool for the Retrieval of Interacting Genes/Proteins
sva	Surrogate Variable Analysis (R/Bioconductor package)
Th1	T Helper 1 cell
Th17	T Helper 17 cell
TSS	Transcription Start Site
UMAP	Uniform Manifold Approximation and Projection
VLA-4	Very Late Antigen-4
WGBS	Whole-Genome Bisulfite Sequencing
WM	White Matter
WML	White Matter Lesion
